# Supplementary material for: A medullary hub for controlling REM sleep and pontine waves
Source: Nat Commun. 2023 Jul 3;14:3922. doi: 10.1038/s41467-023-39496-0 (PMC10318054; doi:10.1038/s41467-023-39496-0)
Supplement: Supplementary file 1 — Supplementary Information [file 41467_2023_39496_MOESM1_ESM.pdf]

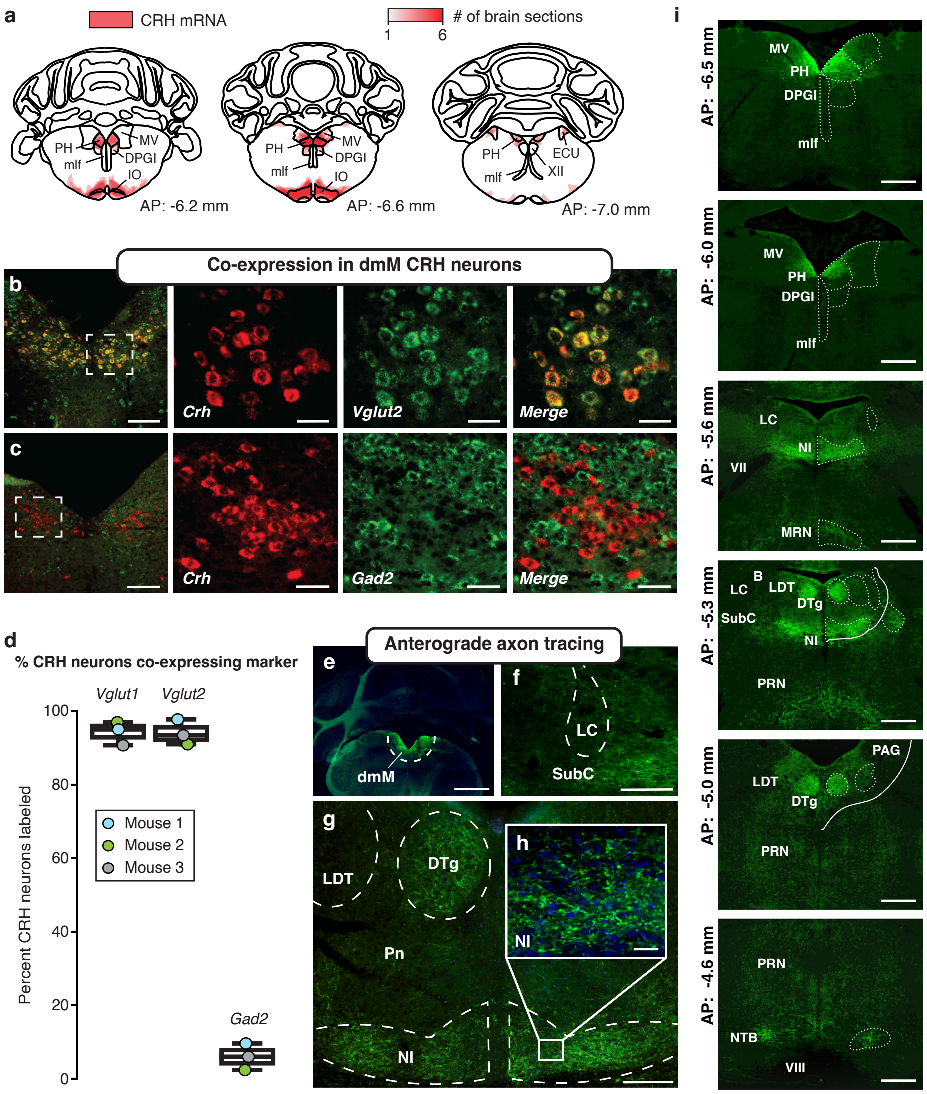

Supplementary Fig. 1: Histological analysis of dmM CRH neuron identity and anatomical projections.

a Outline of areas with cells expressing CRH, generated from brain sections hybridized with a fluorescent probe against *Crh* mRNA. The red color code indicates the number of brain sections containing CRH neurons at the corresponding location (n = 3 mice, 2 brain sections per mouse). Coronal brain schemes adapted from the Allen Reference Atlas – Mouse Brain (atlas.brain-map.org). PH, nucleus prepositus; DPGI, dorsal paragigantocellular nucleus; MV, medial vestibular nucleus; mlf, medial longitudinal fasciculus; IO, inferior olivary complex; ECU, external cuneate nucleus; XII, hypoglossal nucleus.

b-c Localization of fluorescent probes against *Crh* mRNA (red), *Vglut2* (**b**) or *Gad2* (**c**) mRNA (green), and overlay of both channels (yellow). Scale bar, 130 µm (left panel), 30 µm (remaining panels).

d Boxplot showing the percentage of dmM CRH neurons co-expressing *Vglut1* (94.3%), *Vglut2* (94.1%), and *Gad2* (6.2%). Dots, individual mice (n = 3). Center lines represent the median, box limits represent the interquartile range, and whiskers represent the remaining distribution.

e Coronal fluorescence image of dmM in a CRH-Cre mouse expressing ChR2-eYFP for viral tracing. Blue, Hoechst stain. Scale bar, 1.0 mm.

f Fluorescence labeled dmM CRH axons in the dorsolateral pons. Scale bar, 300 μm. LC, locus coeruleus; SubC, subcoeruleus nucleus.

g Fluorescence labeled dmM CRH axons in the dorsal pons. Area outlined by the white box is shown on an expanded scale in panel **h**. Scale bar, 250 μm. DTg, dorsal tegmental nucleus; LDT, laterodorsal tegmental nucleus; NI, nucleus incertus; Pn, pontine central gray.

h Fluorescence labeled dmM CRH axons in the NI, expanded from panel **g**. Blue, Hoechst stain. Scale bar, 100 μm.

i Fluorescence labeled axons of dmM CRH neurons in coronal brain sections along the anteroposterior (AP) axis. Scale bar, 0.5 mm. MRN, magnocellular reticular nucleus; PRN, pontine reticular nucleus; B, Barrington's nucleus; PAG, periaqueductal gray; NTB, nucleus of the trapezoid body; VII, facial nerve; VIII, vestibulocochlear nerve. Fluorescence labeling patterns shown in representative images (**e-i**) were reproduced in n = 3 mice. Source data are provided as a Source Data file.

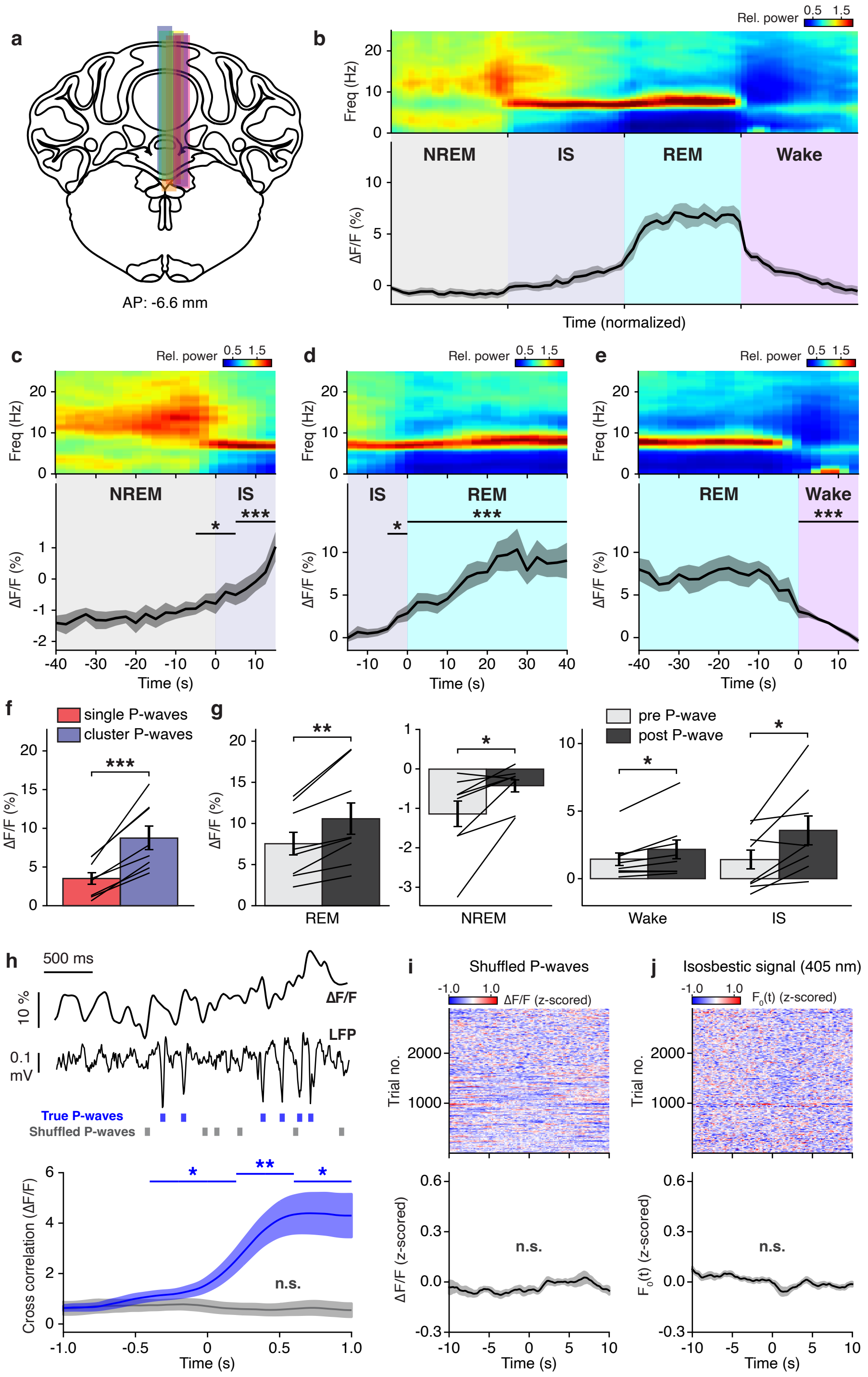

Supplementary Fig. 2: dmM CRH calcium activity during brain state transitions and P-waves.

a Location of optic fiber tracts (colored bars) for fiber photometry recordings in GCaMP6s mice (n = 7 mice).

b Averaged EEG spectrogram (top) and ΔF/F signal (bottom) during NREM→IS→REM→wake transitions (n = 7 mice). The duration of each state episode was normalized in time.

c Averaged EEG spectrogram and ΔF/F activity during NREM→IS transitions (n = 102 transitions from 7 mice).

Baseline interval, -40 to -35 s. Paired t-tests ($P_{-5—0s}$=0.046; $P_{0—5s}$=0.010; $P_{5—10s}$=1.95e-5; $P_{10—15s}$=1.99e-8, Bonferroni-corrected).

d Averaged EEG spectrogram and ΔF/F activity during IS→REM transitions (n = 43 transitions from 7 mice). Baseline interval, -15 to -10 s. Paired t-tests ($P_{-5—0s}$=0.032; $P_{0—5s}$=1.14e-5; $P_{5—10s}$=7.60e-6; $P_{10—15s}$=3.80e-9; $P_{15—20s}$=3.58e-7; $P_{20—25s}$=3.48e-11; $P_{25—30s}$=7.95e-10; $P_{30—35s}$=4.75e-10; $P_{35—40s}$=9.29e-10, Bonferroni-corrected).

e Averaged EEG spectrogram and ΔF/F activity during REM→wake transitions (n = 52 transitions from 7 mice). Baseline interval, -40 to -35 s. Paired t-tests ($P_{0—5s}$=8.45e-8; $P_{5—10s}$=1.79e-9; $P_{10—15s}$=2.81e-11, Bonferroni-corrected).

f ΔF/F activity following single and clustered P-waves (n = 8 mice). Paired t-test (P = 4.34e-4).

g ΔF/F activity preceding vs following P-waves in each brain state (n = 8 mice). Paired t-tests (REM, P = 0.003; NREM, P = 0.026; Wake, P = 0.020; IS, P = 0.022, uncorrected).

h Cross-correlation between ΔF/F activity and P-waves during REM sleep (n = 8 mice). Top, example traces of the ΔF/F signal and pontine LFP. Blue ticks indicate true P-waves, gray ticks indicate time-shuffled P-waves. Bottom, mean normalized cross-correlation (**Methods**) showing the time lag of ΔF/F activity relative to true and time-shuffled P-waves. Baseline interval, -1 to -0.8 s. Paired t-tests ($P_{-0.4 – -2s}$=0.013; $P_{-0.2—0s}$=0.026; $P_{0—0.2s}$=0.023; $P_{0.2—0.4s}$=0.009; $P_{0.4—0.6s}$=0.009; $P_{0.6—0.8s}$=0.018; $P_{0.8—1s}$=0.029, Bonferroni-corrected).

i Z-scored ΔF/F activity surrounding time-shuffled P-waves (n = 8 mice). Top, color-coded ΔF/F signal surrounding each shuffled P-wave (rows); bottom, mean ΔF/F signal.

j Z-scored amplitude of the calcium-independent isosbestic signal surrounding P-waves (n = 8 mice). All statistical comparisons were two-tailed. EEG spectrograms (**b**-**e**) were normalized at each frequency by mean power across the recording. Error bars and shadings indicate ± standard error of the mean (SEM). Lines, individual mice. *P<0.05; **P<0.01; ***P<0.001. Statistical details (**c-j**) shown in **Supplementary Table 1**. Source data are provided as a Source Data file.

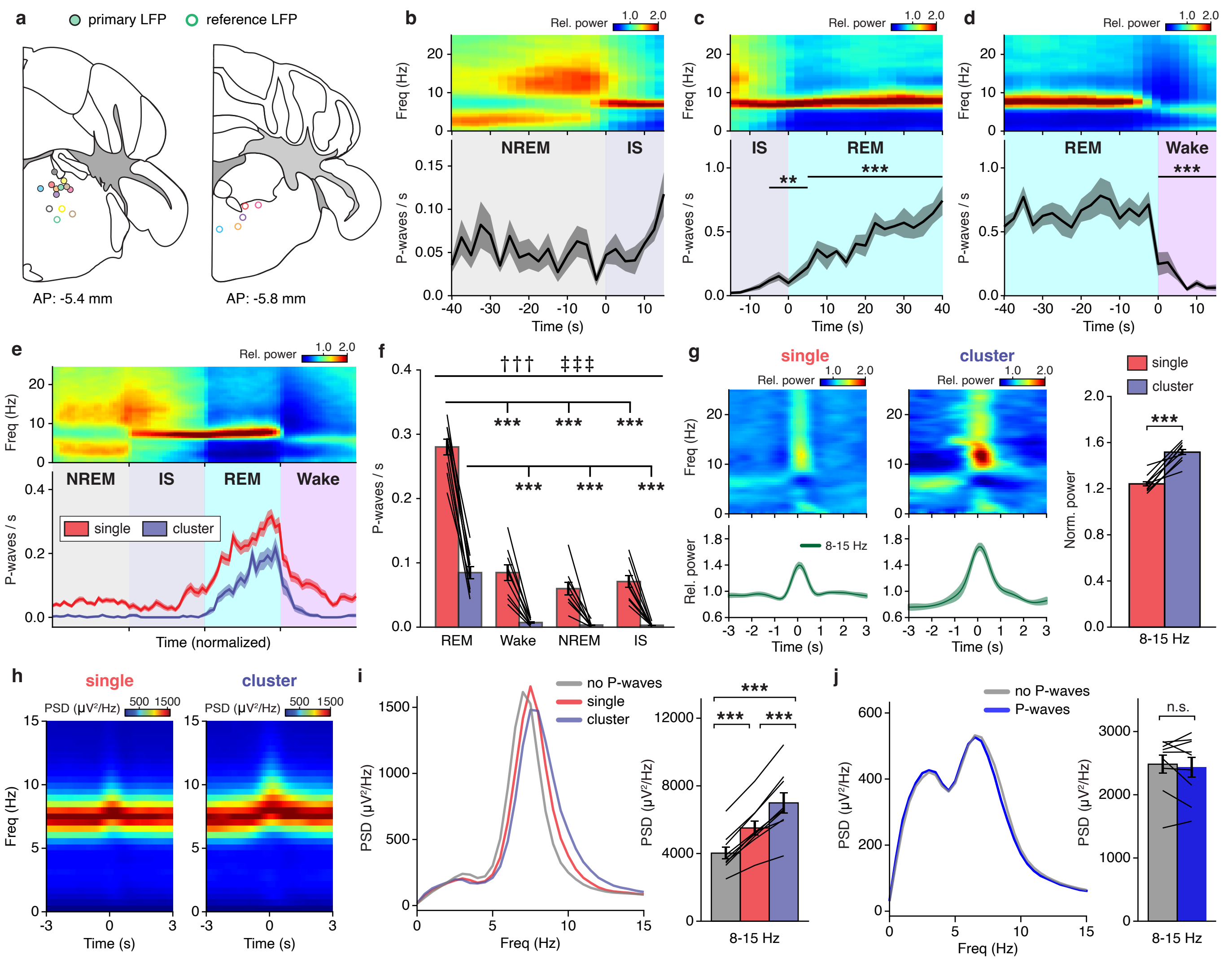

Supplementary Fig. 3: State dependency and EEG correlates of single and clustered P-waves.

a Location of electrode tips for pontine LFP recordings (n = 9 mice). Pairs of circles with the same color represent the two electrode tips for each individual mouse. Filled circles, primary LFP signal; open circles, reference LFP signal.

b Averaged EEG spectrogram and P-wave frequency during NREM→IS transitions (n = 339 transitions from 9 mice). Baseline interval, -40 to -35 s.

c Averaged EEG spectrogram and P-wave frequency during IS→REM transitions (n = 127 transitions from 9 mice). Baseline interval, -15 to -10 s. Paired t-tests ($P_{-5—0s}$=0.003; $P_{0—5s}$=0.002; $P_{5—10s}$=6.80e-08; $P_{10—15s}$=6.41e-10; $P_{15—20s}$=3.36e-9; $P_{20—25s}$=3.10e-10; $P_{25—30s}$=2.44e-14; $P_{30—35s}$=1.90e-12; $P_{35—40s}$=1.27e-13, Bonferroni-corrected).

d Averaged EEG spectrogram and P-wave frequency during REM→wake transitions (n = 157 transitions from 9 mice). Baseline interval, -40 to -35 s. Paired t-tests ($P_{0–5s}$=8.62e-4; $P_{5–10s}$=4.48e-7; $P_{10–15s}$=5.97e-10, Bonferroni-corrected).

e Averaged EEG spectrogram and single vs clustered P-wave frequency during NREM→IS→REM→wake transitions (n = 9 mice). The duration of each state episode was normalized in time.

f Average frequency of single and clustered P-waves per brain state (n = 9 mice). Two-way repeated measures ANOVA (P_{event}= 1.34e-7; $P_{eventXstate}$= 6.74e-8) with Bonferroni post-hoc (single P-waves, REM vs Wake, P = 1.89e-5; REM vs NREM, P = 1.42e-5; REM vs IS, P = 9.92e-6; clustered P-waves, REM vs Wake, P = 3.38e-4; REM vs NREM, P = 2.38e-4; REM vs IS, P = 3.68e-4).

g Left, mean normalized EEG spectrogram and high theta frequency band power (8–15 Hz) surrounding single and clustered P-waves during REM sleep (n = 9 mice). P-waves are aligned at 0 s. Right, mean normalized high theta power during the 1 s interval surrounding single and clustered P-waves. Paired t-test (P = 5.41e-04).

h Averaged raw (i.e. non-normalized) EEG spectrograms surrounding single and clustered P-waves during REM sleep (n = 9 mice).

i Left, mean power spectral density (PSD) of the EEG during the 1 s interval surrounding single and clustered P-waves, as well as during REM sleep epochs without P-waves (n = 9 mice). Right, mean absolute power in the high theta frequency range. One-way repeated measures ANOVA (P = 1.26e-6) with Holm post-hoc (no P-waves vs single P-waves, P = 6.62e-6; no P-waves vs clustered P-waves, P = 4.73e-6; single vs clustered P-waves, P = 5.63e-5).

j Mean PSD (left) and absolute high theta power (right) during wakefulness with and without P-waves (n = 9 mice).

All statistical comparisons were two-tailed. EEG spectrograms in **i** are presented without normalization, while all other spectrograms were normalized at each frequency by mean power across the recording (**b-e**) or across the shown 6 s time interval (**g**). Error bars and shadings indicate ± SEM. Lines, individual mice. † denotes main effect of P-wave type (single vs clustered); ‡ denotes interaction between P-wave type and brain state. **P<0.01; ***P<0.001. Statistical details (**b-d,f,g,i,j**) shown in **Supplementary Table 1**. Source data are provided as a Source Data file.

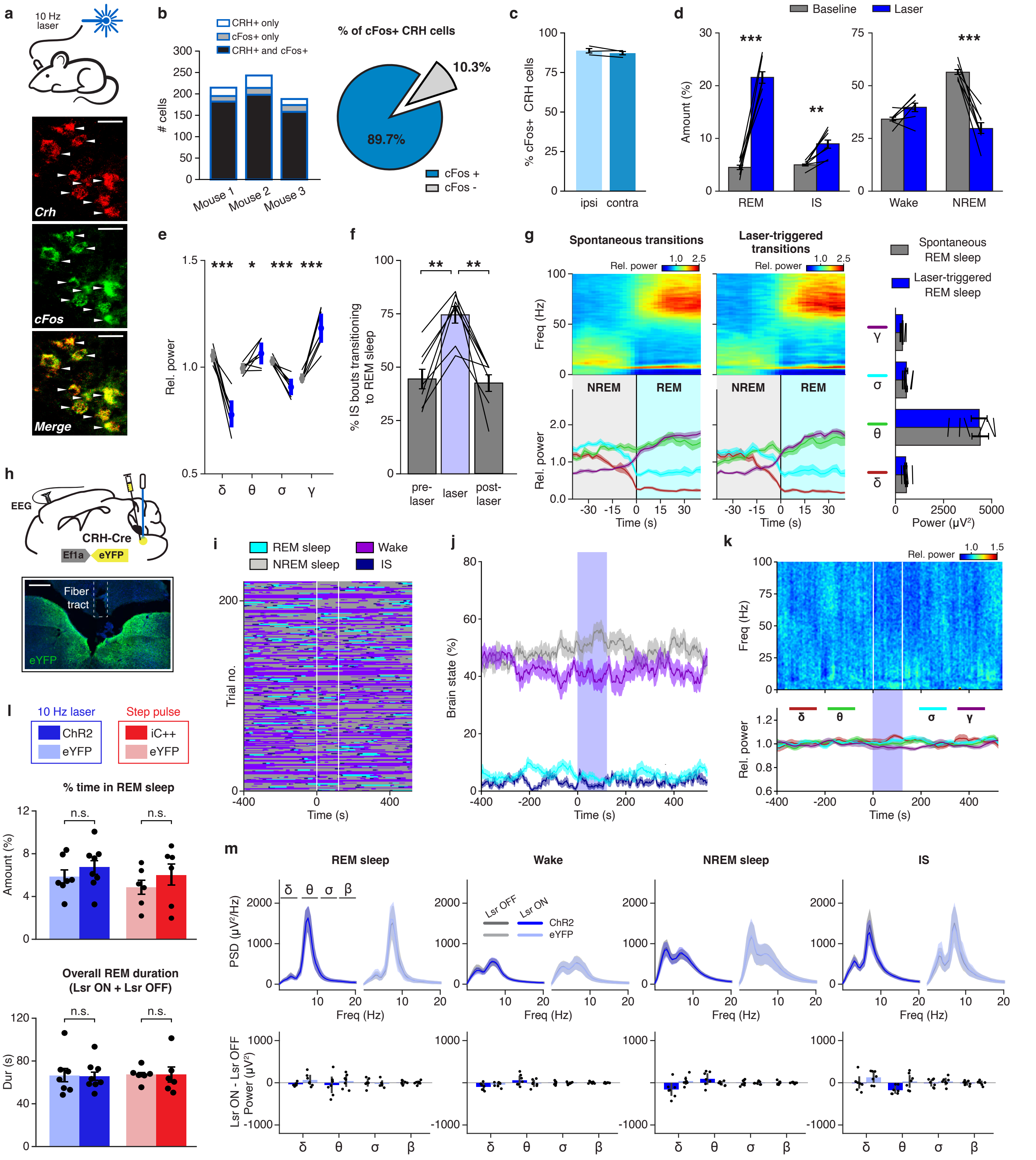

Supplementary Fig. 4: Effects of laser stimulation in mice expressing ChR2-eYFP or eYFP in the dmM.

a cFos expression in the dmM of ChR2 mice (n = 3) following laser stimulation. Top, experimental approach for optogenetically exciting CRH neurons (10 Hz pulse trains, 10 s per minute for 2 hours). Bottom, localization of

fluorescent probes against *Crh* mRNA (red), *cFos* mRNA (green), and overlay of both channels (yellow). Arrowheads, cells co-expressing *Crh* and *cFos*. Scale bars, 20 μm.

b Left, quantification of *Crh* and *cFos* expression for each mouse (n = 3). Right, overall proportion of dmM CRH neurons co-expressing *cFos* mRNA.

c Percentage of *cFos* expressing CRH neurons on the ipsilateral (ipsi) and contralateral (contra) side of the dmM, relative to the optic fiber in ChR2 animals (n = 3).

d Percent time spent in each brain state during the laser stimulation interval and the preceding 120 s baseline interval (n = 7 mice). Paired t-tests (REM, P = 3.00e-05; NREM, P = 1.75e-04; IS, P = 0.004, uncorrected).

e Mean normalized power in the δ (0.5–4 Hz), θ (6–10 Hz), σ (11–15 Hz), and γ (55–99 Hz) frequency bands during the baseline and laser stimulation intervals (n = 7 mice). Paired t-tests (δ, P = 1.96e-4; θ, P = 0.035; σ, P = 3.10e-04; γ, P = 4.52e-04, uncorrected).

f Percentage of IS bouts resulting in a transition to REM sleep during the 120 s intervals preceding, during, and following laser stimulation (n = 7 mice). One-way repeated measures ANOVA (P = 9.52e-4) with Bonferroni post-hoc (laser vs pre-laser, P = 0.004; laser vs post-laser, P = 0.003).

g Left, EEG activity during spontaneous and laser-triggered transitions to REM sleep (n = 7 mice). IS was classified as NREM sleep for this analysis. Top, averaged EEG spectrograms; bottom, normalized power in the δ, θ, σ, and γ frequency bands. Right, mean power in each frequency band during spontaneous vs laser-triggered REM sleep.

h Top, experimental approach for optogenetic control studies. Bottom, coronal fluorescence image of dmM in an eYFP control mouse. Blue, Hoechst stain. Scale bar, 300 μm.

i Behavioral state of eYFP mice surrounding each laser stimulation trial (rows; n = 7 mice).

j Percentages of REM sleep, NREM sleep, IS, and wake surrounding the laser stimulation interval (blue shading; n = 7 mice).

k Impact of laser stimulation on the EEG spectrogram and different power bands in eYFP mice (n = 7). Top, averaged EEG spectrogram; bottom, normalized power in the δ, θ, σ, and γ frequency bands.

l Top, mean overall percentage of REM sleep during closed-loop optogenetic activation (ChR2, n = 8; eYFP, n = 7) and inhibition (iC++, n = 6; eYFP, n = 6) experiments. Bottom, mean overall duration of REM sleep episodes, averaged across both laser-on and laser-off bouts.

m Top, power spectral density (PSD) of the EEG with and without laser stimulation during each brain state for ChR2 (n = 7) and eYFP mice (n = 7). Horizontal lines in the leftmost plot show the frequency ranges of the δ, θ, σ, and β frequency bands. Shadings, ± standard deviation (SD). Bottom, mean difference in the power of each frequency band between laser-on and laser-off brain states. Error bars, 95% confidence interval (CI). EEG spectrograms (**g**,**k**) were normalized at each frequency by mean power across the recording. Error bars and shadings indicate ± SEM unless otherwise stated. Individual mice are represented by lines (**c-g**) or dots (**l**,**m**). *P<0.05; **P<0.01; ***P<0.001. Statistical details (**d-g,l,m**) shown in **Supplementary Table 1**. Source data are provided as a Source Data file.

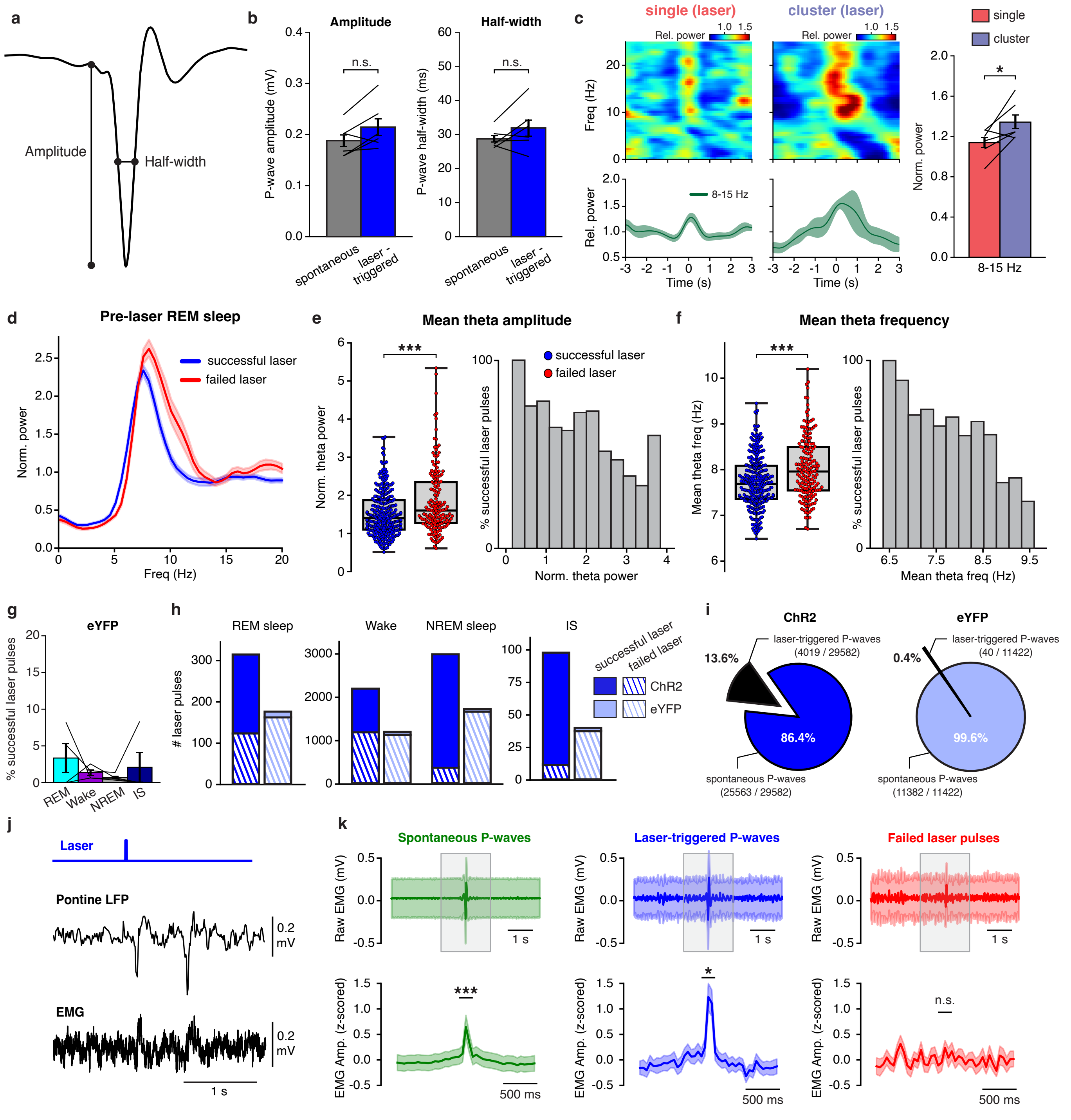

Supplementary Fig. 5: Comparison of laser-triggered P-waves with spontaneous P-waves and failed laser pulses during REM sleep.

a Schematic of P-wave amplitude and half-width measurements.

b Mean amplitude and half-width of spontaneous and laser-triggered P-waves during REM sleep (n = 6 mice).

c Left, mean normalized EEG spectrogram and high theta frequency band power (8–15 Hz) surrounding laser-triggered single and clustered P-waves during REM sleep (n = 6 mice). Each frequency component was

normalized by mean power across the 6 s interval surrounding the P-waves (aligned at 0 s). Right, mean normalized high theta power during the 1 s interval surrounding single and clustered P-waves. Paired t-test (P = 0.047).

d Normalized PSD during the 3 s interval preceding successful and failed laser pulses during REM sleep (n = 6 mice). Each frequency component was normalized by mean power across the recording.

e Left, distribution of normalized theta power (6–12 Hz) preceding successful and failed laser pulses (n = 6 mice). Point-biserial correlation (rpb = -0.223, P = 7.22e-7). Right, probability of laser success by preceding theta power.

f Left, distribution of mean theta frequency preceding successful and failed laser pulses (n = 6 mice). Point-biserial correlation (rpb = -0.214, P = 2.14e-6). Right, probability of laser success by preceding theta frequency.

g Percentage of laser pulses successfully triggering a P-wave for each brain state in eYFP mice (n = 4).

h Number of successful and failed laser pulses per brain state for ChR2 (n = 6) and eYFP mice (n = 4).

i Total number of spontaneous and laser-triggered P-waves for ChR2 (n = 6) and eYFP mice (n = 4).

j Example recording with spontaneous and laser-triggered P-wave, including laser stimulation trial, LFP trace, and EMG trace.

k Top, averaged raw EMG signal (shading, ± SD) surrounding spontaneous P-waves, laser-triggered P-waves, and failed laser pulses during REM sleep (n = 5 mice). Bottom, z-scored amplitude of the EMG signal (**Methods**). Mean EMG amplitude was compared between the 100 ms interval surrounding the P-wave or failed laser pulse and the preceding baseline interval. Paired t-tests (spontaneous P-waves, P = 7.29e-4; laser-triggered P-waves, P = 0.020, uncorrected). For boxplots, center lines represent the median, box limits represent the interquartile range, and whiskers represent the remaining distribution. Error bars and shadings indicate ± SEM unless otherwise stated. Lines, individual mice; dots, individual laser pulses. *P<0.05; ***P<0.001. Statistical details (**b,c,e-g,k**) shown in **Supplementary Table 1**. Source data are provided as a Source Data file.

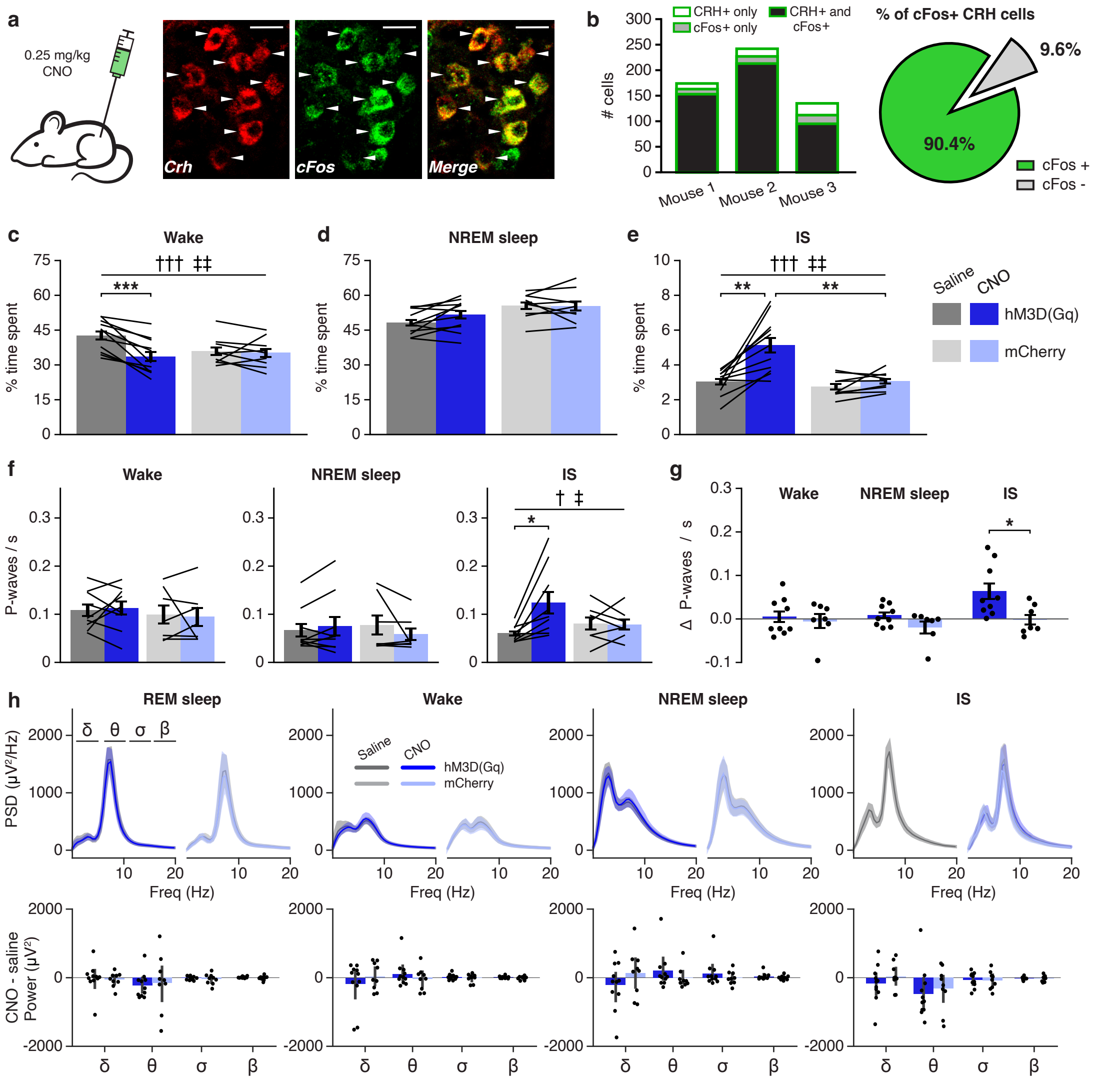

Supplementary Fig. 6: Effects of chemogenetic excitation in mice expressing hM3D(Gq) or mCherry in the dmM.

a cFos expression in the dmM of hM3D(Gq) mice (n = 3) following DREADD stimulation. Left, experimental approach for chemogenetically exciting CRH neurons (i.p. injection of 0.25 mg/kg clozapine-N-oxide (CNO) 2 hours prior to sacrifice). Right, localization of fluorescent probes against *Crh* mRNA (red), *cFos* mRNA (green), and overlay of both channels (yellow). Arrowheads, cells co-expressing *Crh* and *cFos*. Scale bars, 20 μm.

b Left, quantification of *Crh* and *cFos* expression for each mouse (n = 3). Right, overall proportion of dmM CRH neurons co-expressing *cFos* mRNA.

c Percent time spent in wakefulness during saline vs CNO trials in hM3D(Gq) (n = 11) and mCherry mice (n = 9). Mixed ANOVA (P_{drug}= 6.03e-4; $P_{drugXvirus}$= 0.004) with Holm post-hoc (hM3D(Gq), saline vs CNO, P = 7.52e-4).

d Percent time spent in NREM sleep in hM3D(Gq) (n = 11) and mCherry mice (n = 9).

e Percent time spent in IS in hM3D(Gq) (n = 11) and mCherry mice (n = 9). Mixed ANOVA (P_{drug}= 1.30e-4; $P_{drugXvirus}$= 0.003) with Holm post-hoc (hM3D(Gq), saline vs CNO, P = 0.002; CNO, hM3D(Gq) vs mCherry, P = 0.003).

f Mean P-wave frequency during wake, NREM sleep, and IS in hM3D(Gq) (n = 9) and mCherry mice (n = 7). Mixed ANOVA (IS, P_{drug}= 0.014; $P_{drugXvirus}$= 0.023) with Holm post-hoc (hM3D(Gq), saline vs CNO, P = 0.027).

g Mean change in P-wave frequency between CNO and saline trials in hM3D(Gq) (n = 9) vs mCherry mice (n = 7). Unpaired t-tests (IS, P = 0.023, uncorrected).

h Top, PSD of the EEG during saline and CNO trials for each brain state in hM3D(Gq) (n = 11) and mCherry mice (n = 9). Horizontal lines in the leftmost plot show the frequency ranges of the δ, θ, σ, and β frequency bands. Shadings, ± SD. Bottom, mean difference in the power of each frequency band between CNO and saline trials. Error bars, 95% CI. Error bars and shadings indicate ± SEM unless otherwise stated. Individual mice are represented by lines (**c-f**) or dots (**g**,**h**). † denotes main effect of drug (saline vs CNO); ‡ denotes interaction between drug and virus. *P<0.05; **P<0.01; ***P<0.001. Statistical details (**c-h**) shown in **Supplementary Table 1**. Source data are provided as a Source Data file.

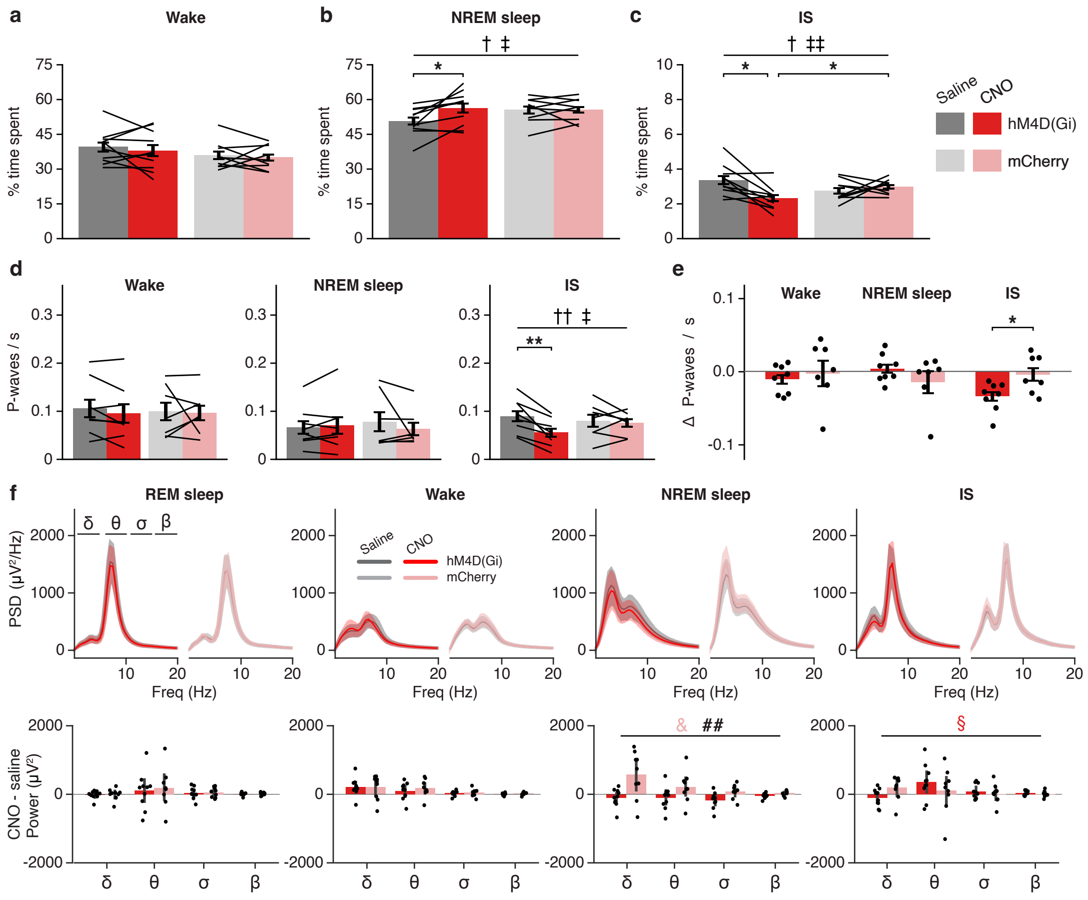

Supplementary Fig. 7: Effects of chemogenetic inhibition in mice expressing hM4D(Gi) or mCherry in the dmM.

a Percent time spent in wakefulness during saline vs CNO trials in hM4D(Gi) (n = 10) and mCherry mice (n = 9).

b Percent time spent in NREM sleep in hM4D(Gi) (n = 10) and mCherry mice (n = 9). Mixed ANOVA (P_{drug}= 0.033; $P_{drugXvirus}$= 0.035) with Holm post-hoc (hM4D(Gi), saline vs CNO, P = 0.035).

c Percent time spent in IS in hM4D(Gi) (n = 10) and mCherry mice (n = 9). Mixed ANOVA (P_{drug}= 0.048; $P_{drugXvirus}$= 0.007) with Holm post-hoc (hM4D(Gi), saline vs CNO, P = 0.012; CNO, hM4D(Gi) vs mCherry, P = 0.045).

d Mean P-wave frequency during wake, NREM sleep, and IS in hM4D(Gi) (n = 8) and mCherry mice (n = 7). Mixed ANOVA (IS, P_{drug}= 0.004; $P_{drugXvirus}$= 0.024) with Holm post-hoc (hM4D(Gi), saline vs CNO, P = 0.004).

e Mean change in P-wave frequency between CNO and saline trials in hM4D(Gi) (n = 8) vs mCherry mice (n = 7). Unpaired t-tests (IS, P = 0.024, uncorrected).

f Top, PSD of the EEG during saline and CNO trials for each brain state in hM4D(Gi) (n = 10) and mCherry mice (n = 9). Horizontal lines in the leftmost plot show the frequency ranges of the δ, θ, σ, and β frequency bands. Shadings, ± SD. Bottom, mean difference in the power of each frequency band between CNO and

saline trials. Error bars, 95% CI. In each brain state, two-way repeated measures ANOVA was used to statistically compare the EEG between saline and CNO trials for hM4D(Gi) mice (IS, P_{drug}= 0.253; $P_{drugXband}$= 0.017) and mCherry mice (NREM, P_{drug}= 0.079; $P_{drugXband}$= 0.024), and mixed ANOVA was used to compare the CNO-induced EEG changes between hM4D(Gi) and mCherry mice (NREM, P_{virus}= 0.108; $P_{virusXband}$= 0.004). Error bars and shadings indicate ± SEM unless otherwise stated. Individual mice are represented by lines (**a-d**) or dots (**e**,**f**). In panels **a-d**, † denotes the main effect of the drug (saline vs CNO), and ‡ denotes the interaction between drug and virus. In panel **f**, § denotes the interaction between drug and frequency band power for hM4D(Gi) mice, & denotes the same for mCherry mice, and # denotes the interaction between virus (hM4D(Gi) vs mCherry) and change in frequency band power. *P<0.05; **P<0.01. Statistical details (**a-f**) shown in **Supplementary Table 1**. Source data are provided as a Source Data file.

Supplementary Table 1

Figure	Pairwise test	Tail	Comparison	T-statistic	P-value	Sig. Level	Group analysis	F-statistic	P-value
1f	post-hoc with Bonferroni correction	two-tailed	REM vs Wake<br>REM vs NREM<br>REM vs IS<br>Wake vs NREM<br>Wake vs IS<br>NREM vs IS	T(6) = 4.60<br>T(6) = 5.47<br>T(6) = 5.58<br>T(6) = 4.17<br>T(6) = 0.13<br>T(6) = 2.50	0.022<br>0.009<br>0.008<br>0.035<br>1.0<br>0.280	*<br>**<br>**<br>*<br>ns<br>ns	One-way repeated measures ANOVA with brain state as the within factor	$F_{(3,18)}$ = 24.59	1.38e-06
2c	post-hoc with Bonferroni correction	two-tailed	REM vs Wake<br>REM vs NREM<br>REM vs IS<br>Wake vs NREM<br>Wake vs IS<br>NREM vs IS	T(8) = 8.84<br>T(8) = 11.94<br>T(8) = 10.89<br>T(8) = 4.95<br>T(8) = 2.10<br>T(8) = 1.77	1.26e-04<br>1.58e-05<br>2.81e-05<br>0.007<br>0.417<br>0.690	***<br>***<br>***<br>**<br>ns<br>ns	One-way repeated measures ANOVA with brain state as the within factor	$F_{(3,24)}$ = 99.25	1.15e-13
2i	repeated paired t-tests with Bonferroni correction	two-tailed	Baseline vs -7.5 to -5 s<br>Baseline vs -5 to -2.5 s<br>Baseline vs -2.5 to 0 s<br>Baseline vs 0 to 2.5 s<br>Baseline vs 2.5 to 5 s<br>Baseline vs 5 to 7.5 s<br>Baseline vs 7.5 to 10 s	T(7) = 0.35<br>T(7) = 0.63<br>T(7) = 2.48<br>T(7) = 10.23<br>T(7) = 7.42<br>T(7) = 1.88<br>T(7) = 1.43	1.0<br>1.0<br>0.296<br>1.29e-04<br>9.27e-04<br>0.717<br>1.0	ns<br>ns<br>ns<br>***<br>***<br>ns<br>ns			
2j	repeated paired t-tests with Bonferroni correction	two-tailed	Baseline vs -0.8 to -0.6 s<br>Baseline vs -0.6 to -0.4 s<br>Baseline vs -0.4 to -0.2 s<br>Baseline vs -0.2 to 0 s<br>Baseline vs 0 to 0.2 s<br>Baseline vs 0.2 to 0.4 s<br>Baseline vs 0.4 to 0.6 s<br>Baseline vs 0.6 to 0.8 s<br>Baseline vs 0.8 to 1 s	T(7) = 2.82<br>T(7) = 3.62<br>T(7) = 4.65<br>T(7) = 6.11<br>T(7) = 5.97<br>T(7) = 6.72<br>T(7) = 11.27<br>T(7) = 10.79<br>T(7) = 10.16	0.232<br>0.077<br>0.021<br>0.004<br>0.005<br>0.002<br>8.70e-05<br>1.16e-04<br>1.73e-04	ns<br>ns<br>*<br>**<br>**<br>**<br>***<br>***<br>***			
2k (top)	repeated paired t-tests with Bonferroni correction	two-tailed	Baseline vs -7.5 to -5 s<br>Baseline vs -5 to -2.5 s<br>Baseline vs -2.5 to 0 s<br>Baseline vs 0 to 2.5 s<br>Baseline vs 2.5 to 5 s<br>Baseline vs 5 to 7.5 s<br>Baseline vs 7.5 to 10 s	T(7) = 0.03<br>T(7) = 0.23<br>T(7) = 0.75<br>T(7) = 6.37<br>T(7) = 5.27<br>T(7) = 1.67<br>T(7) = 1.22	1.0<br>1.0<br>1.0<br>0.003<br>0.008<br>0.976<br>1.0	ns<br>ns<br>ns<br>**<br>**<br>ns<br>ns			
2k (bottom)	repeated paired t-tests with Bonferroni correction	two-tailed	Baseline vs -7.5 to -5 s<br>Baseline vs -5 to -2.5 s<br>Baseline vs -2.5 to 0 s<br>Baseline vs 0 to 2.5 s<br>Baseline vs 2.5 to 5 s<br>Baseline vs 5 to 7.5 s<br>Baseline vs 7.5 to 10 s	T(7) = 0.76<br>T(7) = 0.82<br>T(7) = 1.91<br>T(7) = 7.97<br>T(7) = 4.20<br>T(7) = 1.02<br>T(7) = 0.45	1.0<br>1.0<br>0.680<br>6.54e-04<br>0.028<br>1.0<br>1.0	ns<br>ns<br>ns<br>***<br>*<br>ns<br>ns			
3h (left)	post-hoc with Holm correction	two-tailed	ChR2, laser vs no laser<br>eYFP, laser vs no laser<br><br>No laser, ChR2 vs eYFP<br>Laser, ChR2 vs eYFP	T(7) = 5.35<br>T(6) = 0.40<br><br>T(13) = 1.88<br>T(13) = 1.64	0.002<br>0.700<br><br>0.192<br>0.192	**<br>ns<br><br>ns<br>ns	Mixed ANOVA with laser and virus (ChR2 vs eYFP) as within and between factors	Main effect:<br>$F_{(1,13)}$ = 16.92<br><br>Interaction:<br>$F_{(1,13)}$ = 10.78	p_{laser}<br>0.001<br><br>$p_{laserXvirus}$<br>0.006
3i (left)	post-hoc with Holm correction	two-tailed	iC++, laser vs no laser<br>eYFP, laser vs no laser<br><br>No laser, iC++ vs eYFP<br>Laser, iC++ vs eYFP	T(5) = 4.46<br>T(5) = 0.41<br><br>T(10) = 1.70<br>T(10) = 2.24	0.013<br>0.701<br><br>0.120<br>0.098	*<br>ns<br><br>ns<br>ns	Mixed ANOVA with laser and virus (iC++ vs eYFP) as within and between factors	Main effect:<br>$F_{(1,10)}$ = 12.89<br><br>Interaction:<br>$F_{(1,10)}$ = 9.28	p_{laser}<br>0.005<br><br>$p_{laserXvirus}$<br>0.012
4f	post-hoc with Bonferroni correction	two-tailed	Control vs spon. P-waves<br>Control vs laser P-waves<br>Control vs failed laser<br>Failed laser vs spon. P-waves<br>Failed laser vs laser P-waves<br>Spon. vs laser P-waves	T(5) = 8.43<br>T(5) = 4.97<br>T(5) = 0.27<br>T(5) = 5.20<br>T(5) = 5.46<br>T(5) = 0.05	0.002<br>0.025<br>1.0<br>0.021<br>0.017<br>1.0	**<br>*<br>ns<br>*<br>*<br>ns	One-way repeated measures ANOVA with event type as the within factor	$F_{(3,15)}$ = 21.62	1.06e-05

Figure	Pairwise test	Tail	Comparison	T-statistic	P-value	Sig. Level	Group analysis	F-statistic	P-value
4g (left)	post-hoc with Bonferroni correction	two-tailed	REM vs Wake<br>REM vs NREM<br>REM vs IS<br>Wake vs NREM<br>Wake vs IS<br>NREM vs IS	T(5) = 3.09<br>T(5) = 4.58<br>T(5) = 7.13<br>T(5) = 7.07<br>T(5) = 7.35<br>T(5) = 0.74	0.163<br>0.036<br>0.005<br>0.005<br>0.004<br>1.0	ns<br>*<br>**<br>**<br>**<br>ns	One-way repeated measures ANOVA with brain state as the within factor	$F_{(3,15)}$ = 32.14	8.95e-07
4g (right)	paired t-test	two-tailed	True vs time-shuffled laser	T(5) = 10.49	1.36e-04	***			
5d	post-hocs with Holm correction	two-tailed	*% time spent in REM sleep*<br>hM3D(Gq), saline vs CNO<br>mCherry, saline vs CNO<br>Saline, hM3D(Gq) vs mCherry<br>CNO, hM3D(Gq) vs mCherry	T(10) = 5.76<br>T(8) = 1.04<br>T(18) = 0.35<br>T(18) = 4.44	3.65e-04<br>0.329<br>0.730<br>6.38e-04	***<br>ns<br>ns<br>***	Mixed ANOVAs with drug (saline vs CNO) and virus (hM3D(Gq) vs mCherry) as within and between factors	Main effect: $F_{(1,18)}$ = 29.69<br>Interaction: $F_{(1,18)}$ = 14.77	p_{drug} 3.55e-05<br>$p_{drugXvirus}$ 0.001
5e			*REM sleep frequency*<br>hM3D(Gq), saline vs CNO<br>mCherry, saline vs CNO<br>Saline, hM3D(Gq) vs mCherry<br>CNO, hM3D(Gq) vs mCherry	T(10) = 3.59<br>T(8) = 0.87<br>T(18) = 1.35<br>T(18) = 4.60	0.010<br>0.408<br>0.198<br>0.001	*<br>ns<br>ns<br>**		Main effect: $F_{(1,18)}$ = 13.53<br>Interaction: $F_{(1,18)}$ = 8.80	p_{drug} 0.002<br>$p_{drugXvirus}$ 0.008
5f			*REM sleep duration*<br>—	—	—	—		Main effect: $F_{(1,18)}$ = 0.01<br>Interaction: $F_{(1,18)}$ = 1.50	p_{drug} 0.916<br>$p_{drugXvirus}$ 0.236
5g			*IS-->REM transition prob.*<br>hM3D(Gq), saline vs CNO<br>mCherry, saline vs CNO<br>Saline, hM3D(Gq) vs mCherry<br>CNO, hM3D(Gq) vs mCherry	T(10) = 4.71<br>T(8) = 1.13<br>T(18) = 0.76<br>T(18) = 1.10	0.002<br>0.290<br>0.578<br>0.578	**<br>ns<br>ns<br>ns		Main effect: $F_{(1,18)}$ = 18.69<br>Interaction: $F_{(1,18)}$ = 5.93	p_{drug} 4.10e-04<br>$p_{drugXvirus}$ 0.026
5h	post-hocs with Holm correction	two-tailed	*% time spent in REM sleep*<br>hM4D(Gi), saline vs CNO<br>mCherry, saline vs CNO<br>Saline, hM4D(Gi) vs mCherry<br>CNO, hM4D(Gi) vs mCherry	T(9) = 5.44<br>T(8) = 1.19<br>T(17) = 0.94<br>T(17) = 5.94	8.19e-04<br>0.269<br>0.361<br>3.73e-05	***<br>ns<br>ns<br>***	Mixed ANOVAs with drug (saline vs CNO) and virus (hM4D(Gi) vs mCherry) as within and between factors	Main effect: $F_{(1,17)}$ = 11.51<br>Interaction: $F_{(1,17)}$ = 22.59	p_{drug} 0.003<br>$p_{drugXvirus}$ 1.84e-04
5i			*REM sleep frequency*<br>hM4D(Gi), saline vs CNO<br>mCherry, saline vs CNO<br>Saline, hM4D(Gi) vs mCherry<br>CNO, hM4D(Gi) vs mCherry	T(9) = 4.42<br>T(8) = 0.90<br>T(17) = 1.52<br>T(17) = 2.56	0.003<br>0.396<br>0.155<br>0.041	**<br>ns<br>ns<br>*		Main effect: $F_{(1,17)}$ = 12.13<br>Interaction: $F_{(1,17)}$ = 17.17	p_{drug} 0.003<br>$p_{drugXvirus}$ 6.79e-04
5j			*REM sleep duration*<br>hM4D(Gi), saline vs CNO<br>mCherry, saline vs CNO<br>Saline, hM4D(Gi) vs mCherry<br>CNO, hM4D(Gi) vs mCherry	T(9) = 2.69<br>T(8) = 1.82<br>T(17) = 0.70<br>T(17) = 2.74	0.046<br>0.107<br>0.492<br>0.028	*<br>ns<br>ns<br>*		Main effect: $F_{(1,17)}$ = 0.82<br>Interaction: $F_{(1,17)}$ = 10.17	p_{drug} 0.378<br>$p_{drugXvirus}$ 0.005
5k			*IS-->REM transition prob.*<br>hM4D(Gi), saline vs CNO<br>mCherry, saline vs CNO<br>Saline, hM4D(Gi) vs mCherry<br>CNO, hM4D(Gi) vs mCherry	T(9) = 4.36<br>T(8) = 0.59<br>T(17) = 0.89<br>T(17) = 3.85	0.004<br>0.571<br>0.388<br>0.003	**<br>ns<br>ns<br>**		Main effect: $F_{(1,17)}$ = 6.31<br>Interaction: $F_{(1,17)}$ = 10.59	p_{drug} 0.022<br>$p_{drugXvirus}$ 0.005

Figure	Pairwise test	Tail	Comparison	T-statistic	P-value	Sig. Level	Group analysis	F-statistic	P-value
6c (top)	post-hoc with Holm correction	two-tailed	hM3D(Gq), saline vs CNO<br>mCherry, saline vs CNO<br><br>Saline, hM3D(Gq) vs mCherry<br>CNO, hM3D(Gq) vs mCherry	T(8) = 5.41<br>T(6) = 0.15<br><br>T(14) = 0.04<br>T(14) = 2.01	0.001<br>0.884<br><br>0.970<br>0.129	**<br>ns<br><br>ns<br>ns	Mixed ANOVA with drug and virus (hM3D(Gq) vs mCherry) as within and between factors	Main effect:<br>$F_{(1,14)}$ = 11.95<br><br>Interaction:<br>$F_{(1,14)}$ = 7.88	p_{drug}<br>0.004<br><br>$p_{drugXvirus}$<br>0.014
6e (top)	post-hoc with Holm correction	two-tailed	hM4D(Gi), saline vs CNO<br>mCherry, saline vs CNO<br><br>Saline, hM4D(Gi) vs mCherry<br>CNO, hM4D(Gi) vs mCherry	T(7) = 2.90<br>T(6) = 0.25<br><br>T(13) = 0.15<br>T(13) = 3.70	0.046<br>0.810<br><br>0.833<br>0.010	*<br>ns<br><br>ns<br>*	Mixed ANOVA with drug and virus (hM4D(Gi) vs mCherry) as within and between factors	Main effect:<br>$F_{(1,13)}$ = 7.00<br><br>Interaction:<br>$F_{(1,13)}$ = 4.96	p_{drug}<br>0.020<br><br>$p_{drugXvirus}$<br>0.044
Supp. 2c	repeated paired t-tests with Bonferroni correction	two-tailed	Baseline vs -35 to -30 s<br>Baseline vs -30 to -25 s<br>Baseline vs -25 to -20 s<br>Baseline vs -20 to -15 s<br>Baseline vs -15 to -10 s<br>Baseline vs -10 to -5 s<br>Baseline vs -5 to 0 s<br>Baseline vs 0 to 5 s<br>Baseline vs 5 to 10 s<br>Baseline vs 10 to 15 s	T(6) = 1.30<br>T(6) = 0.40<br>T(6) = 1.16<br>T(6) = 1.34<br>T(6) = 2.11<br>T(6) = 1.95<br>T(6) = 2.93<br>T(6) = 3.42<br>T(6) = 5.07<br>T(6) = 6.61	1.0<br>1.0<br>1.0<br>1.0<br>0.415<br>0.588<br>0.046<br>0.010<br>1.95e-05<br>1.99e-08	ns<br>ns<br>ns<br>ns<br>ns<br>ns<br>*<br>*<br>***<br>***			
Supp. 2d	repeated paired t-tests with Bonferroni correction	two-tailed	Baseline vs -10 to -5 s<br>Baseline vs -5 to 0 s<br>Baseline vs 0 to 5 s<br>Baseline vs 5 to 10 s<br>Baseline vs 10 to 15 s<br>Baseline vs 15 to 20 s<br>Baseline vs 20 to 25 s<br>Baseline vs 25 to 30 s<br>Baseline vs 30 to 35 s<br>Baseline vs 35 to 40 s	T(6) = 0.53<br>T(6) = 3.16<br>T(6) = 5.71<br>T(6) = 5.83<br>T(6) = 8.15<br>T(6) = 6.76<br>T(6) = 9.66<br>T(6) = 8.64<br>T(6) = 8.81<br>T(6) = 8.59	1.0<br>0.032<br>1.14e-05<br>7.60e-06<br>3.80e-09<br>3.58e-07<br>3.48e-11<br>7.95e-10<br>4.75e-10<br>9.29e-10	ns<br>*<br>***<br>***<br>***<br>***<br>***<br>***<br>***<br>***			
Supp. 2e	repeated paired t-tests with Bonferroni correction	two-tailed	Baseline vs -35 to -30 s<br>Baseline vs -30 to -25 s<br>Baseline vs -25 to -20 s<br>Baseline vs -20 to -15 s<br>Baseline vs -15 to -10 s<br>Baseline vs -10 to -5 s<br>Baseline vs -5 to 0 s<br>Baseline vs 0 to 5 s<br>Baseline vs 5 to 10 s<br>Baseline vs 10 to 15 s	T(6) = 2.05<br>T(6) = 0.85<br>T(6) = 0.49<br>T(6) = 0.32<br>T(6) = 0.52<br>T(6) = 0.28<br>T(6) = 2.56<br>T(6) = 6.91<br>T(6) = 7.97<br>T(6) = 9.14	0.497<br>1.0<br>1.0<br>1.0<br>1.0<br>1.0<br>1.0<br>8.45e-08<br>1.79e-09<br>2.81e-11	ns<br>ns<br>ns<br>ns<br>ns<br>ns<br>ns<br>***<br>***<br>***			
Supp. 2f	paired t-test	two-tailed	Single vs cluster P-waves	T(7) = 6.08	4.34e-04	***			
Supp. 2g	paired t-tests without correction	two-tailed	REM, pre vs post-P-wave<br>Wake, pre vs post-P-wave<br>NREM, pre vs post-P-wave<br>IS, pre vs post-P-wave	T(7) = 4.45<br>T(7) = 2.81<br>T(7) = 2.99<br>T(7) = 2.94	0.003<br>0.026<br>0.020<br>0.022	**<br>*<br>*<br>*			
Supp. 2h	repeated paired t-tests with Bonferroni correction	two-tailed	<u>*True P-waves*</u><br>Baseline vs -0.8 to -0.6 s<br>Baseline vs -0.6 to -0.4 s<br>Baseline vs -0.4 to -0.2 s<br>Baseline vs -0.2 to 0 s<br>Baseline vs 0 to 0.2 s<br>Baseline vs 0.2 to 0.4 s<br>Baseline vs 0.4 to 0.6 s<br>Baseline vs 0.6 to 0.8 s<br>Baseline vs 0.8 to 1 s<br><br><u>*Time-shuffled P-waves*</u><br>Baseline vs -0.8 to -0.6 s<br>Baseline vs -0.6 to -0.4 s<br>Baseline vs -0.4 to -0.2 s<br>Baseline vs -0.2 to 0 s<br>Baseline vs 0 to 0.2 s	 T(7) = 1.12<br>T(7) = 2.64<br>T(7) = 5.06<br>T(7) = 4.49<br>T(7) = 4.59<br>T(7) = 5.39<br>T(7) = 5.37<br>T(7) = 4.80<br>T(7) = 4.39<br><br><br>T(7) = 2.06<br>T(7) = 1.08<br>T(7) = 0.82<br>T(7) = 0.62<br>T(7) = 0.06	 1.0<br>0.302<br>0.013<br>0.026<br>0.023<br>0.009<br>0.009<br>0.018<br>0.029<br><br><br>0.710<br>1.0<br>1.0<br>1.0<br>1.0	 ns<br>ns<br>*<br>*<br>*<br>**<br>**<br>*<br>*<br><br><br>ns<br>ns<br>ns<br>ns<br>ns			

Figure	Pairwise test	Tail	Comparison	T-statistic	P-value	Sig. Level	Group analysis	F-statistic	P-value
			Baseline vs 0.2 to 0.4 s<br>Baseline vs 0.4 to 0.6 s<br>Baseline vs 0.6 to 0.8 s<br>Baseline vs 0.8 to 1 s	T(7) = 0.40<br>T(7) = 0.50<br>T(7) = 0.07<br>T(7) = 0.27	1.0<br>1.0<br>1.0<br>1.0	ns<br>ns<br>ns<br>ns			
Supp. 2i	repeated paired t-tests with Bonferroni correction	two-tailed	Baseline vs -7.5 to -5 s<br>Baseline vs -5 to -2.5 s<br>Baseline vs -2.5 to 0 s<br>Baseline vs 0 to 2.5 s<br>Baseline vs 2.5 to 5 s<br>Baseline vs 5 to 7.5 s<br>Baseline vs 7.5 to 10 s	T(7) = 0.58<br>T(7) = 0.55<br>T(7) = 0.03<br>T(7) = 0.02<br>T(7) = 0.07<br>T(7) = 0.46<br>T(7) = 0.65	1.0<br>1.0<br>1.0<br>1.0<br>1.0<br>1.0<br>1.0	ns<br>ns<br>ns<br>ns<br>ns<br>ns<br>ns			
Supp. 2j	repeated paired t-tests with Bonferroni correction	two-tailed	Baseline vs -7.5 to -5 s<br>Baseline vs -5 to -2.5 s<br>Baseline vs -2.5 to 0 s<br>Baseline vs 0 to 2.5 s<br>Baseline vs 2.5 to 5 s<br>Baseline vs 5 to 7.5 s<br>Baseline vs 7.5 to 10 s	T(7) = 0.04<br>T(7) = 1.86<br>T(7) = 2.25<br>T(7) = 3.60<br>T(7) = 3.30<br>T(7) = 3.74<br>T(7) = 3.68	1.0<br>0.742<br>0.414<br>0.061<br>0.092<br>0.054<br>0.058	ns<br>ns<br>ns<br>ns<br>ns<br>ns<br>ns			
Supp. 3b	repeated paired t-tests with Bonferroni correction	two-tailed	Baseline vs -35 to -30 s<br>Baseline vs -30 to -25 s<br>Baseline vs -25 to -20 s<br>Baseline vs -20 to -15 s<br>Baseline vs -15 to -10 s<br>Baseline vs -10 to -5 s<br>Baseline vs -5 to 0 s<br>Baseline vs 0 to 5 s<br>Baseline vs 5 to 10 s<br>Baseline vs 10 to 15 s	T(8) = 0.69<br>T(8) = 0.28<br>T(8) = 0.76<br>T(8) = 1.96<br>T(8) = 2.42<br>T(8) = 1.73<br>T(8) = 1.97<br>T(8) = 0.83<br>T(8) = 1.82<br>T(8) = 0.36	1.0<br>1.0<br>1.0<br>0.562<br>0.178<br>0.928<br>0.547<br>1.0<br>0.759<br>1.0	ns<br>ns<br>ns<br>ns<br>ns<br>ns<br>ns<br>ns<br>ns<br>ns			
Supp. 3c	repeated paired t-tests with Bonferroni correction	two-tailed	Baseline vs -10 to -5 s<br>Baseline vs -5 to 0 s<br>Baseline vs 0 to 5 s<br>Baseline vs 5 to 10 s<br>Baseline vs 10 to 15 s<br>Baseline vs 15 to 20 s<br>Baseline vs 20 to 25 s<br>Baseline vs 25 to 30 s<br>Baseline vs 30 to 35 s<br>Baseline vs 35 to 40 s	T(8) = 2.07<br>T(8) = 3.78<br>T(8) = 3.89<br>T(8) = 6.19<br>T(8) = 7.61<br>T(8) = 6.56<br>T(8) = 7.90<br>T(8) = 8.82<br>T(8) = 7.75<br>T(8) = 8.40	0.447<br>0.003<br>0.002<br>6.80e-08<br>6.41e-10<br>3.36e09<br>3.10e-10<br>2.44e-14<br>1.90e-12<br>1.27e-13	ns<br>**<br>**<br>***<br>***<br>***<br>***<br>***<br>***<br>***			
Supp. 3d	repeated paired t-tests with Bonferroni correction	two-tailed	Baseline vs -35 to -30 s<br>Baseline vs -30 to -25 s<br>Baseline vs -25 to -20 s<br>Baseline vs -20 to -15 s<br>Baseline vs -15 to -10 s<br>Baseline vs -10 to -5 s<br>Baseline vs -5 to 0 s<br>Baseline vs 0 to 5 s<br>Baseline vs 5 to 10 s<br>Baseline vs 10 to 15 s	T(8) = 1.31<br>T(8) = 0.20<br>T(8) = 0.98<br>T(8) = 0.82<br>T(8) = 2.14<br>T(8) = 1.41<br>T(8) = 0.80<br>T(8) = 4.05<br>T(8) = 6.67<br>T(8) = 6.87	1.0<br>1.0<br>1.0<br>1.0<br>0.370<br>1.0<br>1.0<br>8.62e-04<br>4.48e-07<br>5.97e-10	ns<br>ns<br>ns<br>ns<br>ns<br>ns<br>ns<br>***<br>***<br>***			
Supp. 3f	post-hoc with Bonferroni correction	two-tailed	Single P-waves, REM vs Wake<br>Single P-waves, REM vs NREM<br>Single P-waves, REM vs IS<br>Single P-waves, Wake vs NREM<br>Single P-waves, Wake vs IS<br>Single P-waves, NREM vs IS<br>Cluster P-waves, REM vs Wake<br>Cluster P-waves, REM vs NREM<br>Cluster P-waves, REM vs IS<br>Cluster P-waves, Wake vs NREM<br>Cluster P-waves, Wake vs IS<br>Cluster P-waves, NREM vs IS	T(8) = 12.20<br>T(8) = 12.82<br>T(8) = 13.59<br>T(8) = 1.48<br>T(8) = 0.75<br>T(8) = 1.34<br>T(8) = 8.04<br>T(8) = 8.57<br>T(8) = 7.80<br>T(8) = 2.47<br>T(8) = 1.95<br>T(8) = 0.24	1.89-05<br>1.42e-05<br>9.92e-06<br>0.704<br>0.950<br>0.704<br>3.38e-04<br>2.38e-04<br>3.68e-04<br>0.232<br>0.435<br>0.950	***<br>***<br>***<br>ns<br>ns<br>ns<br>***<br>***<br>***<br>ns<br>ns<br>ns	Two-way repeated measures ANOVA with event (single vs cluster P-waves) and brain state as the within factors	Main effect:<br>$F_{(1,8)}$ = 295.33<br><br>Interaction:<br>$F_{(3,24)}$ = 44.05	p_{event}<br>1.34e-07<br><br>$p_{eventXstate}$<br>6.74e-08
Supp. 3g	paired t-test	two-tailed	Single vs cluster P-waves	T(8) = 5.55	5.41e-04	***			

Figure	Pairwise test	Tail	Comparison	T-statistic	P-value	Sig. Level	Group analysis	F-statistic	P-value
Supp. 3i	post-hoc with Holm correction	two-tailed	No P-waves vs single P-waves<br>No P-waves vs cluster P-waves<br>Single vs cluster P-waves	T(8) = 11.95<br>T(8) = 10.81<br>T(8) = 7.72	6.62e-06<br>4.73e-06<br>5.63e-05	***<br>***<br>***	One-way repeated measures ANOVA with event as the within factor	$F_{(2,16)}$ = 103.48	1.26e-06
Supp. 3j	paired t-test	two-tailed	No P-waves vs P-waves	T(8) = 0.67	0.525	ns			
Supp. 4d	paired t-tests without correction	two-tailed	REM, baseline vs laser<br>Wake, baseline vs laser<br>NREM, baseline vs laser<br>IS, baseline vs laser	T(6) = 11.22<br>T(6) = 2.23<br>T(6) = 8.22<br>T(6) = 4.48	3.00e-05<br>0.068<br>1.75e-04<br>0.004	***<br>ns<br>***<br>**			
Supp. 4e	paired t-tests without correction	two-tailed	δ, baseline vs laser<br>θ, baseline vs laser<br>σ, baseline vs laser<br>γ, baseline vs laser	T(6) = 8.06<br>T(6) = 2.71<br>T(6) = 7.41<br>T(6) = 6.92	1.96e-04<br>0.035<br>3.10e-04<br>4.52e-04	***<br>*<br>***<br>***			
Supp. 4f	post-hoc with Bonferroni correction	two-tailed	Laser vs pre-laser interval<br>Laser vs post-laser interval<br>Pre vs post-laser interval	T(6) = 5.57<br>T(6) = 5.85<br>T(6) = 0.72	0.004<br>0.003<br>1.0	**<br>**<br>ns	One-way repeated measures ANOVA with time interval as the within factor	$F_{(2,12)}$ = 13.13	9.52e-04
Supp. 4g	—	—	—	—	—	—	Two-way repeated measures ANOVA with laser and frequency band as the within factors	Main effect:<br>$F_{(1,6)}$ = 0.47<br><br>Interaction:<br>$F_{(3,18)}$ = 0.40	p_{laser}<br>0.519<br><br>$p_{laserXband}$<br>0.584
Supp. 4l (top)	unpaired t-tests without correction	two-tailed	ChR2 vs eYFP<br>iC++ vs eYFP	T(13) = 0.89<br>T(13) = 0.82	0.388<br>0.432	ns<br>ns			
Supp. 4l (bottom)	unpaired t-tests without correction	two-tailed	ChR2 vs eYFP<br>iC++ vs eYFP	T(13) = 0.06<br>T(13) = 0.04	0.952<br>0.973	ns<br>ns			
Supp. 4m	—	—	—	—	—	—	Two-way repeated measures ANOVAs with laser and frequency band as the within factors	***ChR2***<br>*REM sleep*<br>Main effect:<br>$F_{(1,6)}$ = 0.40<br>Interaction:<br>$F_{(3,18)}$ = 0.39<br><br>*Wake*<br>Main effect:<br>$F_{(1,6)}$ = 0.11<br>Interaction:<br>$F_{(3,18)}$ = 3.69<br><br>*NREM sleep*<br>Main effect:<br>$F_{(1,6)}$ = 1.83<br>Interaction:<br>$F_{(3,18)}$ = 3.40<br><br>*IS*<br>Main effect:<br>$F_{(1,6)}$ = 4.47<br>Interaction:<br>$F_{(3,18)}$ = 3.33<br><br>***eYFP***<br>*REM sleep*<br>Main effect:<br>$F_{(1,6)}$ = 0.45<br>Interaction:<br>$F_{(3,18)}$ = 1.04	p_{laser}<br>0.555<br>$p_{laserXband}$<br>0.581<br><br>p_{laser}<br>0.748<br>$p_{laserXband}$<br>0.096<br><br>p_{laser}<br>0.224<br>$p_{laserXband}$<br>0.112<br><br>p_{laser}<br>0.079<br>$p_{laserXband}$<br>0.078<br><br>p_{laser}<br>0.527<br>$p_{laserXband}$<br>0.379

Figure	Pairwise test	Tail	Comparison	T-statistic	P-value	Sig. Level	Group analysis	F-statistic	P-value
								<u>*Wake*</u><br>Main effect:<br>$F_{(1,6)}$ = 1.58<br>Interaction:<br>$F_{(3,18)}$ = 0.96<br><br><u>*NREM sleep*</u><br>Main effect:<br>$F_{(1,6)}$ = 0.17<br>Interaction:<br>$F_{(3,18)}$ = 1.19<br><br><u>*IS*</u><br>Main effect:<br>$F_{(1,6)}$ = 1.36<br>Interaction:<br>$F_{(3,18)}$ = 2.52	 p_{laser}<br>0.256<br>$p_{laserXband}$<br>0.398<br><br><br>p_{laser}<br>0.696<br>$p_{laserXband}$<br>0.322<br><br><br>p_{laser}<br>0.288<br>$p_{laserXband}$<br>0.118
Supp. 5b (left)	paired t-test	two-tailed	Spon. vs laser P-waves	T(5) = 2.12	0.088	ns			
Supp. 5b (right)	paired t-test	two-tailed	Spon. vs laser P-waves	T(5) = 1.47	0.201	ns			
Supp. 5c	paired t-test	two-tailed	Single vs cluster laser P-waves	T(5) = 2.63	0.047	*			
Supp. 5e	point-biserial correlation	two-tailed	Successful vs failed laser	rpb = -0.223	7.22e-07	***			
Supp. 5f	point-biserial correlation	two-tailed	Successful vs failed laser	rpb = -0.214	2.14e-06	***			
Supp. 5g	—	—	—	—	—	—	One-way repeated measures ANOVA with brain state as the within factor	$F_{(3,9)}$ = 0.72	0.563
Supp. 5k	paired t-tests without correction	two-tailed	Spon. P-waves, baseline vs event<br>Laser P-waves, baseline vs event<br>Failed laser, baseline vs event	T(4) = 9.35<br>T(4) = 3.77<br>T(4) = 0.62	7.29e-04<br>0.020<br>0.569	***<br>*<br>ns			
Supp. 6c<br><br><br><br><br><br><br><br>Supp. 6d<br><br><br><br><br><br><br><br>Supp. 6e	post-hocs with Holm correction	two-tailed	<u>*% time spent in wake*</u><br>hM3D(Gq), saline vs CNO<br>mCherry, saline vs CNO<br><br>Saline, hM3D(Gq) vs mCherry<br>CNO, hM3D(Gq) vs mCherry<br><br><br><u>*% time spent in NREM sleep*</u><br><br><br>—<br><br><br><br><br><u>*% time spent in IS*</u><br>hM3D(Gq), saline vs CNO<br>mCherry, saline vs CNO<br><br>Saline, hM3D(Gq) vs mCherry<br>CNO, hM3D(Gq) vs mCherry	 T(10) = 5.25<br>T(8) = 0.33<br><br>T(18) = 2.32<br>T(18) = 0.53<br><br><br><br><br><br>—<br><br><br><br><br><br>T(10) = 4.60<br>T(8) = 1.57<br><br>T(18) = 0.97<br>T(18) = 4.09	 7.52e-04<br>0.748<br><br>0.065<br>0.601<br><br><br><br><br><br>—<br><br><br><br><br><br>0.002<br>0.156<br><br>0.344<br>0.003	 ***<br>ns<br><br>ns<br>ns<br><br><br><br><br><br>—<br><br><br><br><br><br>**<br>ns<br><br>ns<br>**	Mixed ANOVAs with drug (saline vs CNO) and virus (hM3D(Gq) vs mCherry) as within and between factors	 Main effect:<br>$F_{(1,18)}$ = 17.22<br><br>Interaction:<br>$F_{(1,18)}$ = 10.96<br><br><br><br>Main effect:<br>$F_{(1,18)}$ = 2.27<br><br>Interaction:<br>$F_{(1,18)}$ = 2.26<br><br><br><br>Main effect:<br>$F_{(1,18)}$ = 23.47<br><br>Interaction:<br>$F_{(1,18)}$ = 11.38	 p_{drug}<br>6.03e-04<br><br>$p_{drugXvirus}$<br>0.004<br><br><br><br>p_{drug}<br>0.150<br><br>$p_{drugXvirus}$<br>0.150<br><br><br><br>p_{drug}<br>1.30e-04<br><br>$p_{drugXvirus}$<br>0.003

Figure	Pairwise test	Tail	Comparison	T-statistic	P-value	Sig. Level	Group analysis	F-statistic	P-value
Supp. 6f	post-hocs with Holm correction	two-tailed	*<u>Wake</u>*<br>—<br><br>*<u>NREM sleep</u>*<br>—<br><br>*<u>IS</u>*<br>hM3D(Gq), saline vs CNO<br>mCherry, saline vs CNO<br><br>Saline, hM3D(Gq) vs mCherry<br>CNO, hM3D(Gq) vs mCherry	—<br><br>—<br><br>T(8) = 3.16<br>T(6) = 0.09<br><br>T(14) = 1.30<br>T(14) = 1.68	—<br><br>—<br><br>0.027<br>0.929<br><br>0.239<br>0.239	—<br><br>—<br><br>*<br>ns<br><br>ns<br>ns	Mixed ANOVAs with drug and virus (hM3D(Gq) vs mCherry) as within and between factors	Main effect:<br>$F_{(1,14)}$ = 0.01<br>Interaction:<br>$F_{(1,14)}$ = 0.23<br><br>Main effect:<br>$F_{(1,14)}$ = 0.19<br>Interaction:<br>$F_{(1,14)}$ = 2.87<br><br>Main effect:<br>$F_{(1,14)}$ = 7.80<br>Interaction:<br>$F_{(1,14)}$ = 6.47	p_{drug}<br>0.936<br>$p_{drugXvirus}$<br>0.640<br><br>p_{drug}<br>0.670<br>$p_{drugXvirus}$<br>0.113<br><br>p_{drug}<br>0.014<br>$p_{drugXvirus}$<br>0.023
Supp. 6g	unpaired t-tests without correction	two-tailed	Wake, hM3D(Gq) vs mCherry<br>NREM, hM3D(Gq) vs mCherry<br>IS, hM3D(Gq) vs mCherry	T(14) = 0.48<br>T(14) = 1.69<br>T(14) = 2.54	0.640<br>0.113<br>0.023	ns<br>ns<br>*			
Supp. 6h	—	—	—	—	—	—	Two-way repeated measures ANOVAs with drug and frequency band as the within factors	***hM3D(Gq)***<br>*<u>REM sleep</u>*<br>Main effect:<br>$F_{(1,10)}$ = 2.01<br>Interaction:<br>$F_{(3,30)}$ = 2.32<br><br>*<u>Wake</u>*<br>Main effect:<br>$F_{(1,10)}$ = 0.01<br>Interaction:<br>$F_{(3,30)}$ = 1.14<br><br>*<u>NREM sleep</u>*<br>Main effect:<br>$F_{(1,10)}$ = 0.35<br>Interaction:<br>$F_{(3,30)}$ = 1.45<br><br>*<u>IS</u>*<br>Main effect:<br>$F_{(1,10)}$ = 4.25<br>Interaction:<br>$F_{(3,30)}$ = 3.06<br><br>***mCherry***<br>*<u>REM sleep</u>*<br>Main effect:<br>$F_{(1,8)}$ = 0.38<br>Interaction:<br>$F_{(3,24)}$ = 0.24<br><br>*<u>Wake</u>*<br>Main effect:<br>$F_{(1,8)}$ = 0.11<br>Interaction:<br>$F_{(3,24)}$ = 0.09<br><br>*<u>NREM sleep</u>*<br>Main effect:<br>$F_{(1,8)}$ = 0.05<br>Interaction:<br>$F_{(3,24)}$ = 0.60	 <br>p_{drug}<br>0.186<br>$p_{drugXband}$<br>0.132<br><br><br>p_{drug}<br>0.940<br>$p_{drugXband}$<br>0.350<br><br><br>p_{drug}<br>0.568<br>$p_{drugXband}$<br>0.260<br><br><br>p_{drug}<br>0.066<br>$p_{drugXband}$<br>0.077<br><br><br><br>p_{drug}<br>0.553<br>$p_{drugXband}$<br>0.646<br><br><br>p_{drug}<br>0.751<br>$p_{drugXband}$<br>0.902<br><br><br>p_{drug}<br>0.829<br>$p_{drugXband}$<br>0.491

Figure	Pairwise test	Tail	Comparison	T-statistic	P-value	Sig. Level	Group analysis	F-statistic	P-value
								IS<br>Main effect:<br>$F_{(1,8)} = 0.78$<br>Interaction:<br>$F_{(3,24)} = 2.78$	p_{drug}<br>0.402<br>$p_{drugXband}$<br>0.126
							Mixed ANOVAs with frequency band and virus (hM3D(Gq) vs mCherry) as within and between factors	*REM sleep*<br>Main effect:<br>$F_{(1,18)} = 0.00$<br>Interaction:<br>$F_{(3,54)} = 0.18$	p_{virus}<br>0.982<br>$p_{virusXband}$<br>0.909
								Wake<br>Main effect:<br>$F_{(1,18)} = 0.04$<br>Interaction:<br>$F_{(3,54)} = 0.96$	p_{virus}<br>0.840<br>$p_{virusXband}$<br>0.418
								NREM sleep<br>Main effect:<br>$F_{(1,18)} = 0.05$<br>Interaction:<br>$F_{(3,54)} = 1.76$	p_{virus}<br>0.830<br>$p_{virusXband}$<br>0.166
								IS<br>Main effect:<br>$F_{(1,18)} = 0.42$<br>Interaction:<br>$F_{(3,54)} = 0.39$	p_{virus}<br>0.525<br>$p_{virusXband}$<br>0.763
Supp. 7a	post-hocs with Holm correction	two-tailed	*% time spent in wake*<br>—	—	—	—	Mixed ANOVAs with drug (saline vs CNO) and virus (hM4D(Gi) vs mCherry) as within and between factors	Main effect:<br>$F_{(1,17)} = 0.55$<br>Interaction:<br>$F_{(1,17)} = 0.07$	p_{drug}<br>0.470<br>$p_{drugXvirus}$<br>0.801
Supp. 7b			*% time spent in NREM sleep*<br>hM4D(Gi), saline vs CNO<br>mCherry, saline vs CNO<br>Saline, hM4D(Gi) vs mCherry<br>CNO, hM4D(Gi) vs mCherry	T(9) = 2.91<br>T(8) = 0.08<br>T(17) = 1.85<br>T(17) = 0.30	0.035<br>0.939<br>0.165<br>0.771	*<br>ns<br>ns<br>ns		Main effect:<br>$F_{(1,17)} = 5.40$<br>Interaction:<br>$F_{(1,17)} = 5.27$	p_{drug}<br>0.033<br>$p_{drugXvirus}$<br>0.035
Supp. 7c			*% time spent in IS*<br>hM4D(Gi), saline vs CNO<br>mCherry, saline vs CNO<br>Saline, hM4D(Gi) vs mCherry<br>CNO, hM4D(Gi) vs mCherry	T(9) = 3.58<br>T(8) = 0.76<br>T(17) = 1.73<br>T(17) = 2.54	0.012<br>0.467<br>0.102<br>0.045	*<br>ns<br>ns<br>*		Main effect:<br>$F_{(1,17)} = 4.55$<br>Interaction:<br>$F_{(1,17)} = 9.31$	p_{drug}<br>0.048<br>$p_{drugXvirus}$<br>0.007
Supp. 7d	post-hocs with Holm correction	two-tailed	*Wake*<br>—	—	—	—	Mixed ANOVAs with drug and virus (hM4D(Gi) vs mCherry) as within and between factors	Main effect:<br>$F_{(1,13)} = 0.84$<br>Interaction:<br>$F_{(1,13)} = 0.20$	p_{drug}<br>0.375<br>$p_{drugXvirus}$<br>0.662
			NREM sleep<br>—	—	—	—		Main effect:<br>$F_{(1,13)} = 0.38$<br>Interaction:<br>$F_{(1,13)} = 1.18$	p_{drug}<br>0.547<br>$p_{drugXvirus}$<br>0.297

Figure	Pairwise test	Tail	Comparison	T-statistic	P-value	Sig. Level	Group analysis	F-statistic	P-value
			IS<br>hM4D(Gi), saline vs CNO<br>mCherry, saline vs CNO<br><br>Saline, hM4D(Gi) vs mCherry<br>CNO, hM4D(Gi) vs mCherry	 T(7) = 4.83<br>T(6) = 0.44<br><br>T(13) = 0.52<br>T(13) = 1.55	 0.004<br>0.676<br><br>0.615<br>0.293	 **<br>ns<br><br>ns<br>ns		Main effect:<br>$F_{(1,13)} = 11.89$<br><br>Interaction:<br>$F_{(1,13)} = 6.51$	p_{drug}<br>0.004<br><br>$p_{drugXvirus}$<br>0.024
Supp. 7e	unpaired t-tests without correction	two-tailed	Wake, hM4D(Gi) vs mCherry<br>NREM, hM4D(Gi) vs mCherry<br>IS, hM4D(Gi) vs mCherry	T(13) = 0.45<br>T(13) = 1.09<br>T(13) = 2.55	0.662<br>0.297<br>0.024	ns<br>ns<br>*			
Supp. 7f	post-hocs with Bonferroni correction	two-tailed	*REM sleep*<br>—	—	—	—	Two-way repeated measures ANOVAs with drug and frequency band as the within factors	***hM4D(Gi)***<br>*REM sleep*<br>Main effect:<br>$F_{(1,9)} = 0.30$<br>Interaction:<br>$F_{(3,27)} = 0.44$	p_{drug}<br>0.596<br>$p_{drugXband}$<br>0.530
			Wake<br>—	—	—	—		*Wake*<br>Main effect:<br>$F_{(1,9)} = 1.91$<br>Interaction:<br>$F_{(3,27)} = 2.36$	p_{drug}<br>0.327<br>$p_{drugXband}$<br>0.142
			NREM sleep<br>—	—	—	—		*NREM sleep*<br>Main effect:<br>$F_{(1,9)} = 2.60$<br>Interaction:<br>$F_{(3,27)} = 0.51$	p_{drug}<br>0.141<br>$p_{drugXband}$<br>0.582
			IS<br>δ, saline vs CNO<br>θ, saline vs CNO<br>σ, saline vs CNO<br>β, saline vs CNO	 T(9) = 1.53<br>T(9) = 2.18<br>T(9) = 1.07<br>T(9) = 1.41	 0.637<br>0.228<br>1.0<br>0.767	 ns<br>ns<br>ns<br>ns		*IS*<br>Main effect:<br>$F_{(1,9)} = 1.49$<br>Interaction:<br>$F_{(3,27)} = 7.67$	p_{drug}<br>0.253<br>$p_{drugXband}$<br>0.017
			REM sleep<br>—	—	—	—		***mCherry***<br>*REM sleep*<br>Main effect:<br>$F_{(1,8)} = 0.95$<br>Interaction:<br>$F_{(3,24)} = 0.10$	p_{drug}<br>0.358<br>$p_{drugXband}$<br>0.353
			Wake<br>—	—	—	—		*Wake*<br>Main effect:<br>$F_{(1,8)} = 3.00$<br>Interaction:<br>$F_{(3,24)} = 2.99$	p_{drug}<br>0.121<br>$p_{drugXband}$<br>0.088
			NREM sleep<br>δ, saline vs CNO<br>θ, saline vs CNO<br>σ, saline vs CNO<br>β, saline vs CNO	 T(8) = 2.60<br>T(8) = 1.34<br>T(8) = 1.05<br>T(8) = 1.23	 0.127<br>0.865<br>1.0<br>1.0	 ns<br>ns<br>ns<br>ns		*NREM sleep*<br>Main effect:<br>$F_{(1,8)} = 4.04$<br>Interaction:<br>$F_{(3,24)} = 5.89$	p_{drug}<br>0.079<br>$p_{drugXband}$<br>0.024
			IS<br>—	—	—	—		*IS*<br>Main effect:<br>$F_{(1,8)} = 0.56$<br>Interaction:<br>$F_{(3,24)} = 0.87$	p_{drug}<br>0.477<br>$p_{drugXband}$<br>0.391
			REM sleep<br>—	—	—	—	Mixed ANOVAs with frequency band and virus (hM4D(Gi) vs mCherry) as within	*REM sleep*<br>Main effect:<br>$F_{(1,17)} = 0.11$<br>Interaction:<br>$F_{(3,51)} = 0.10$	p_{virus}<br>0.744<br>$p_{virusXband}$<br>0.959

Figure	Pairwise test	Tail	Comparison	T-statistic	P-value	Sig. Level	Group analysis	F-statistic	P-value
			Wake<br><br>—<br><br>*NREM sleep*<br>δ, hM4D(Gi) vs mCherry<br>θ, hM4D(Gi) vs mCherry<br>σ, hM4D(Gi) vs mCherry<br>β, hM4D(Gi) vs mCherry<br><br>*IS*<br><br>—	 <br>—<br><br><br>T(17) = 2.86<br>T(17) = 1.70<br>T(17) = 2.48<br>T(17) = 2.25<br><br><br><br>—	 <br>—<br><br><br>0.069<br>0.439<br>0.096<br>0.152<br><br><br><br>—	 <br>—<br><br><br>ns<br>ns<br>ns<br>ns<br><br><br><br>—	and between factors	*Wake*<br>Main effect:<br>$F_{(1,17)} = 0.02$<br>Interaction:<br>$F_{(3,51)} = 0.59$<br><br>*NREM sleep*<br>Main effect:<br>$F_{(1,17)} = 3.39$<br>Interaction:<br>$F_{(3,51)} = 5.06$<br><br>*IS*<br>Main effect:<br>$F_{(1,17)} = 0.01$<br>Interaction:<br>$F_{(3,51)} = 3.67$	 p_{virus}<br>0.877<br>$p_{virusXband}$<br>0.627<br><br><br>p_{virus}<br>0.108<br>$p_{virusXband}$<br>0.004<br><br><br>p_{virus}<br>0.951<br>$p_{virusXband}$<br>0.081
