# Supplementary material for: A medullary hub for controlling REM sleep and pontine waves
Source: Nat Commun. 2023 Jul 3;14:3922. doi: 10.1038/s41467-023-39496-0 (PMC10318054; doi:10.1038/s41467-023-39496-0)
Supplement: Supplementary file 3 — Reporting Summary [file 41467_2023_39496_MOESM3_ESM.pdf]

nature portfolio

Corresponding author(s): Dr. Franz Weber

Last updated by author(s): May 10, 2023

Reporting Summary

Nature Portfolio wishes to improve the reproducibility of the work that we publish. This form provides structure for consistency and transparency in reporting. For further information on Nature Portfolio policies, see our Editorial Policies and the Editorial Policy Checklist.

Statistics

For all statistical analyses, confirm that the following items are present in the figure legend, table legend, main text, or Methods section.

n/a	Confirmed	
☐	☒	The exact sample size (*n*) for each experimental group/condition, given as a discrete number and unit of measurement
☐	☒	A statement on whether measurements were taken from distinct samples or whether the same sample was measured repeatedly
☐	☒	The statistical test(s) used AND whether they are one- or two-sided<br>*Only common tests should be described solely by name; describe more complex techniques in the Methods section.*
☒	☐	A description of all covariates tested
☐	☒	A description of any assumptions or corrections, such as tests of normality and adjustment for multiple comparisons
☐	☒	A full description of the statistical parameters including central tendency (e.g. means) or other basic estimates (e.g. regression coefficient) AND variation (e.g. standard deviation) or associated estimates of uncertainty (e.g. confidence intervals)
☐	☒	For null hypothesis testing, the test statistic (e.g. *F*, *t*, *r*) with confidence intervals, effect sizes, degrees of freedom and *P* value noted<br>*Give P values as exact values whenever suitable.*
☒	☐	For Bayesian analysis, information on the choice of priors and Markov chain Monte Carlo settings
☒	☐	For hierarchical and complex designs, identification of the appropriate level for tests and full reporting of outcomes
☒	☐	Estimates of effect sizes (e.g. Cohen's *d*, Pearson's *r*), indicating how they were calculated

Our web collection on statistics for biologists contains articles on many of the points above.

Software and code

Policy information about availability of computer code

Data collection	EEG/EMG/LFP data were recorded using RHD2000 interface software (Intan Technologies, version 1.5.2), controlled using a custom-built Python user interface (Python version 3.7.3; https://github.com/justin0bk/sleepRecording_v9), and lasers were controlled by a Raspberry Pi using custom Python code (https://github.com/justin0bk/socketrecv). Fiber photometry recordings were carried out using the software Synapse (Tucker-Davis Technologies, version 88).
Data analysis	All data were analyzed using custom Python code, publicly available in the GitHub repository at https://doi.org/10.5281/zenodo.7921731 and submitted in a compressed zip file along with an example dataset and demo code. Statistical analyses were performed using the open source Python packages scipy.stats (https://scipy.org/, version 1.6.2) and statsmodels.stats (https://statsmodels.org/, version 0.12.2). Code for sleep recordings is available on GitHub at https://github.com/justin0bk/sleepRecording_v9, and code for sleep annotation, data analysis, and plotting the graphs in the figures is available at https://github.com/fear-the-kraken/Schott_etal_2023.

For manuscripts utilizing custom algorithms or software that are central to the research but not yet described in published literature, software must be made available to editors and reviewers. We strongly encourage code deposition in a community repository (e.g. GitHub). See the Nature Portfolio guidelines for submitting code & software for further information.

Data

Policy information about availability of data

All manuscripts must include a data availability statement. This statement should provide the following information, where applicable:

- Accession codes, unique identifiers, or web links for publicly available datasets
- A description of any restrictions on data availability
- For clinical datasets or third party data, please ensure that the statement adheres to our policy

All processed data necessary to interpret, verify, and extend the results of this study are available in the Source Data file. Due to the large size of the collected EEG and electrophysiology data, raw recordings will be provided by the corresponding author within 2-4 weeks of request.

Initial examination of gene expression patterns was performed using the Allen Mouse Brain Atlas [ISH data] (available from mouse.brain-map.org). Coronal brain schemes were adapted from the Allen Reference Atlas - Mouse Brain (available from atlas.brain-map.org).

Human research participants

Policy information about studies involving human research participants and Sex and Gender in Research.

Reporting on sex and gender	N/A
Population characteristics	N/A
Recruitment	N/A
Ethics oversight	N/A

Note that full information on the approval of the study protocol must also be provided in the manuscript.

Field-specific reporting

Please select the one below that is the best fit for your research. If you are not sure, read the appropriate sections before making your selection.

☒ Life sciences ☐ Behavioural & social sciences ☐ Ecological, evolutionary & environmental sciences

For a reference copy of the document with all sections, see nature.com/documents/nr-reporting-summary-flat.pdf

Life sciences study design

All studies must disclose on these points even when the disclosure is negative.

Sample size	We did not predetermine sample sizes, but cohorts were similarly sized as in other relevant sleep studies (Yu et al., 2019, 'GABA and glutamate neurons in the VTA regulate sleep and wakefulness.' Nature Neuroscience; Gutierrez Herrera et al., 2019, 'Neurons in the Nucleus papilio contribute to the control of eye movements during REM sleep.' Nature Communications).
Data exclusions	Mice with no virus expression, where virus expression was outside the target site or where the optic fiber was misplaced were excluded from the data set. For P-wave recordings, we excluded mice in which neither electrode tip was in the subcoeruleus, as no P-waves could be detected in these animals, and we excluded recordings with significant artifacts in the primary LFP channel. For fiber photometry experiments, we excluded recordings with no REM sleep periods, as well as recordings containing a sudden shift in baseline fluorescence signal, as described in Methods. For all polysomnographic recordings, we excluded recordings with strong EEG artifacts.
Replication	Sleep recordings were repeated multiple times (on separate days) for the same animal to ensure that key findings are reproducible within individuals. Fiber photometry and optogenetics experiments were replicated at least two times, and chemogenetic experiments were replicated two times each for the saline and CNO treatment conditions. Due to the limited number of recording systems, experiments were run using several (3-6) cohorts of mice. Key results were consistent across different cohorts. Anatomical findings from FISH and axon tracing experiments were replicated in three animals using at least two independently performed assays.
Randomization	Mice were randomly assigned to experimental groups, with a roughly equal number of males and females in each group. For optogenetic open-loop stimulation, the timing of laser stimulation was randomized. In closed-loop stimulation, the laser was turned on for randomly selected 50% of REM bouts. For chemogenetic experiments, the order of saline vs. CNO injection days was randomly assigned.
Blinding	The behavioral experiments were automated; investigators were not blinded to group allocation because the same individuals were involved in all stages of the experiment (i.e. performing the initial surgeries, running the experiments, and analyzing the ensuing datasets). However, investigators were blinded to the timing of the laser when annotating open and closed-loop optogenetics recordings, and blinded to the drug condition when annotating chemogenetics recordings.

Reporting for specific materials, systems and methods

We require information from authors about some types of materials, experimental systems and methods used in many studies. Here, indicate whether each material, system or method listed is relevant to your study. If you are not sure if a list item applies to your research, read the appropriate section before selecting a response.

Materials & experimental systems

n/a	Involved in the study	
☐	☒	Antibodies
☒	☐	Eukaryotic cell lines
☒	☐	Palaeontology and archaeology
☐	☒	Animals and other organisms
☒	☐	Clinical data
☒	☐	Dual use research of concern

Methods

n/a	Involved in the study	
☒	☐	ChIP-seq
☒	☐	Flow cytometry
☒	☐	MRI-based neuroimaging

Antibodies

Antibodies used	anti-GFP chicken antibody (1:1000, Aves Lab, GFP-1020)<br>anti-chicken-AlexaFluor488 (1:500, Jackson Immuno Research Laboratories, Inc., 703-545-155)
Validation	All antibodies have been used and validated in previous studies (e.g., Yuan et al., 2016, 'Levels of Cocaine- and Amphetamine-Regulated Transcript in Vagal Afferents in the Mouse Are Unaltered in Response to Metabolic Challenges.' eNeuro)

Animals and other research organisms

Policy information about studies involving animals; ARRIVE guidelines recommended for reporting animal research, and Sex and Gender in Research

Laboratory animals	All experiments were performed in adult male C57BL/6J mice (Jackson Laboratory; stock no. 000664) and male and female CRH-IRES-Cre mice (stock no. 012704) which were aged 6 – 12 weeks old at the point of surgery. Mice were housed in ventilated cages in groups of two to five, and single-housed while recovering from surgery. Food and water were available ad libitum under a 12:12 hour light:dark cycle with light on from 07:00 to 19:00. The colony room was maintained at an ambient temperature of 20 – 23°C and humidity of 40 – 60%.
Wild animals	This study did not involve wild animals.
Reporting on sex	We designed our study to include a balanced representation of male and female mice, and our results thus apply to both sexes. Sex differences were not the focus of this study, and males did not obviously differ from females in the dataset; as such, we did not perform sex-based analyses. The number of male and female mice used for each experiment is provided in the Source Data file.
Field-collected samples	This study did not involve samples collected from the field.
Ethics oversight	All animal care and experimental procedures were approved by the Institutional Animal Care and Use Committee (IACUC) at the University of Pennsylvania and conducted in accordance with the National Institutes of Health Office of Laboratory Animal Welfare Policy.

Note that full information on the approval of the study protocol must also be provided in the manuscript.
